# An acidic medium‐compatible deep‐near‐infrared dye for in vivo imaging

**DOI:** 10.1002/smo.20230001

**Published:** 2023-05-16

**Authors:** Yan Dong, Ye Zou, Xiaotong Jia, Lei Yin, Weiwei He, Xiao Luo, Xuhong Qian, Youjun Yang

**Affiliations:** ^1^ School of Pharmacy East China University of Science and Technology Shanghai China

**Keywords:** acidic medium, gastrointestinal tract, In vivo imaging, near infrared fluorophore

## Abstract

In vivo imaging in the deep near‐infrared (NIR) spectral region, that is, beyond 800 nm, has become popular due to its penetration depth. While imaging of the neutral medium/tissue has been repeatedly showcased, imaging of the high‐acidic medium remains challenging partly because of the high‐lying HOMO orbital and hence a high pK_a_ of the electron‐donating group of the NIR fluorophores. We devised a novel electron‐donating group (**D6**) with which we further synthesized **ECJ**. **ECJ** exhibits an absorption wavelength beyond 900 nm and is fluorescent. Its pK_a_ was found to be lower than zero, rendering it suitable for bioimaging of a highly‐acidic medium. Its potential for practical applications was showcased in proof‐of‐concept in vivo imaging with a mouse model.

## Graphical abstract

Highly acidic mediums with a pH value below 2 are biorelevant. Yet, dyes do not typically fluoresce at such a lower pH, rendering bioimaging in an acidic medium a challenging task. Following the physical organic rationale, we designed a new electron‐donating headgroup (D5) with which push–pull dyes with negative pK_a_ values could be developed. We further constructed a deep‐near‐infrared absorbing/emitting fluorophore for in vivo imaging of acidic mediums.

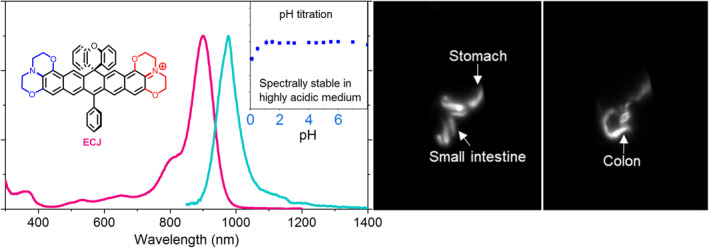

## 1 INTRODUCTION

The pH of the biological fluid is tightly governed and fluctuates in a narrow physiological range. Though the near‐neutral pH is ubiquitous, the acidic medium is not uncommon.[^1^, ^2^, ^3^] Subcellularly, the lysosome has an acidic microenvironment in the pH range from 3.8 to 5.0 to facilitate the degradation of biomacromolecules.[^4^, ^5^, ^6^, ^7^] Endosome experiences a continuous decrease in luminal pH upon endocytosis.[^8^, ^9^, ^10^] The tissue undergoing aerobic glycolysis or hypoxia typically exhibits an acidic microenvironment in pH 6.5–6.8, which promotes tumor growth, metastasis, and drug resistance.[^11^, ^12^, ^13^] Besides, other diseased areas, like inflammation loci^[14]^ and ischemia‐reperfusion injury.^[15]^ Up to the organ level, the skin pH is acidic, which is a biological defense against pathogens.^[16]^ The gastric tract is where the highest acidic medium is found in vivo. The gastric fluid with a pH of 0.9–1.8 is found in the stomach, facilitates digestion, and contributes to antimicrobial defense.[^17^, ^18^, ^19^] Near‐infrared (NIR) fluorescence imaging is a viable approach in disease diagnosis, which allows the direct observation of dynamic biological interests at variant acidic components by organic dyes.[^20^, ^21^, ^22^, ^23^] Yet, the low pH is a major barrier for biological imaging as their donor groups are susceptible to protonation, resulting in a hypochromic shift and fluorescent quenching.[^24^, ^25^] Therefore, acidic medium‐compatible organic fluorescent dyes are a useful addition to the field.

The electronic push–pull (D‐π‐A) scaffold is a general structural theme for organic dyes. For D‐π‐A dyes, the donor groups are typically electron‐donating motifs such as hydroxyl and alkylamino groups.^[26]^ The pK_a_ values of aniline and phenol are at ∼5 and ∼10.0, respectively. When incorporated into the structure of dyes, their pK_a_ decreased due to the electron‐withdrawing effect of an acceptor. However, the pK_a_ value of these dyes are still not low enough to cover the extreme acidity of gastric fluid.[^27^, ^28^, ^29^, ^30^] Consequently, there is an ongoing endeavor to reduce the pK_a_ value through molecular engineering strategy. First, the donor group could be replaced with a rigid julolidine structure, the nitrogen atom of which is forced to participate in hyperconjugation with the aromatic moiety and exhibits a reduced pK_a_.^[31]^ Inductive effects could also be harnessed to lower the pK_a_, such as by incorporating electron‐negative atoms, for example, oxygen or F in the proximity of the nitrogen,^[32]^ for example, morpholine group.^[33]^ The downside is a sequential blue‐shift of the absorption and emission maximum. According to the perturbation theory, the existence of electron‐donating groups at the sites with a large fraction of HOMO orbital leads to a decrease in the bandgap.^[34]^ These considerations lead to a unique design of electron‐donating headgroup (**D6**), which has two benzylic carbons of the julolidine unit replaced by oxygen atoms. It is expected to lower the pK_a_ of the nitrogen atoms via an inductive effect and simultaneously lower the bandgap according to the perturbation theory.

Herein, we first synthesized a series of 2‐styrylindolium derivatives, which is a classic D‐π‐A dyes scaffold, with various alkylamino donating groups to test our hypothesis. After spectral examination and pH titration, we find compound **St6** owned the donating group by installing an oxygen atom into the julolidine group (o‐julolidine), which has the longest emissive wavelength and lowest pK_a_ value. Further, we use this **D6** headgroup to synthesize a bright deep‐NIR fluorochrome (**ECJ**), a close analog of **ECX**.^[31]^ **ECJ** absorbs at 901 nm in CH_2_Cl_2_ and exhibits a negative pK_a_. Finally, we showcased proof‐of‐concept in vivo real‐time gastrointestinal imaging with **ECJ**.

## 2 RESULTS AND DISCUSSION

**FIGURE 1:**
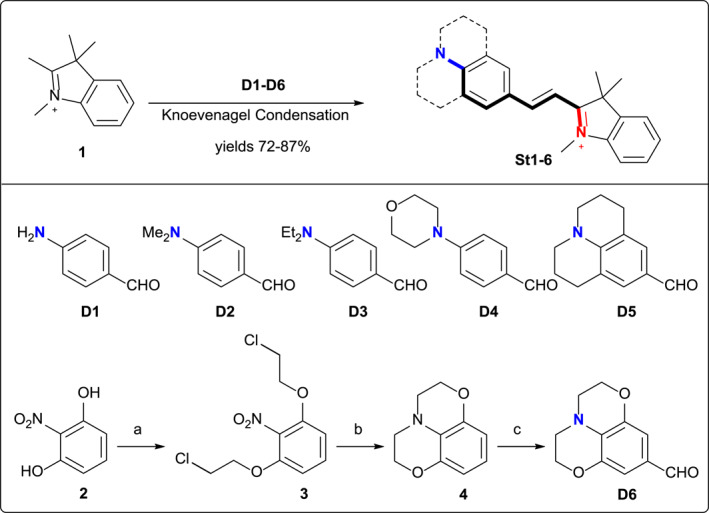
Synthesis of **St1–6**. Reagents and conditions: (a) 1‐bromo‐2‐chloroethane, K_2_CO_3_, CH_3_CN, 90°C, 48 h, 97%; (b) Fe, AcOH, rt −80°C, 6 h, 84%; and (c) POCl_3_, DMF, DCE, reflux, 8 h, 90%.

We prepared six 2‐styrylindolium dyes (**St1–6**) with different electron‐donating headgroups (**D1–D6**) via the Knoevenagel condensation of indolium iodide (**1**) and differently substituted 4‐amino‐benzaldehyde (Figure 1). **St6** was the compound‐of‐interest to verify whether its unique headgroup would impart the desired resistance toward low pH, comparing to **St1**–**St5** bearing common electron‐donating headgroups. **D1**–**D5** were known compounds and **D6** was prepared in three steps starting from the commercial 2‐nitroresorcinol (**2**). Compound **2** was first alkylated with dichloroethane in a 97% yield to give 2,6‐di‐(2′‐chloroethyl)nitrobenzene (**3**), which was subjected to a reductive alkylation condition to furnish **4** in one step. **D6** was obtained by the Vilsmeier–Haack formylation of **4** exclusively para to the nitrogen.

**FIGURE 2:**
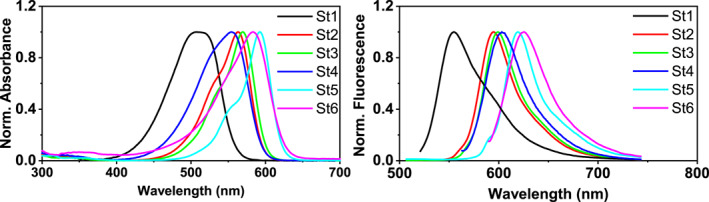
The UV‐Vis absorption and fluorescence emission spectra of **St1–6** in CH_2_Cl_2_.

The UV‐Vis absorption and fluorescence emission spectra of **D1**–**D6** were collected in CH_2_Cl_2_ (Figure 2, Table S1). The absorption maxima of **St1–5** were located at 508, 563, 570, 554, and 592 nm. Such a trend was also in agreement with the existing structure–property relationship in dye chemistry. The absorption maximum of **St5** was at 583 nm, which was 9 nm shorter than that of **St6,** bearing a julolidine headgroup and longer than those of **St1**–**4**. The maximal emission wavelength of **St6** was interestingly longer than those of **St1–5**. Consequently, **ST6** exhibited a remarkably larger Stokes shift. The Stokes shift of a fluorophore is often associated with a decrease in fluorescence brightness. We found that the brightness of **St6**, that is, 1.86 × 10^4^ cm^−1^ M^−1^, was also comparable to **St5**, that is, 1.39 × 10^4^ cm^−1^ M^−1^. These spectral studies verified that **D6** as a headgroup does not induce a notable blue‐shift compared to julolidine.

**FIGURE 3:**
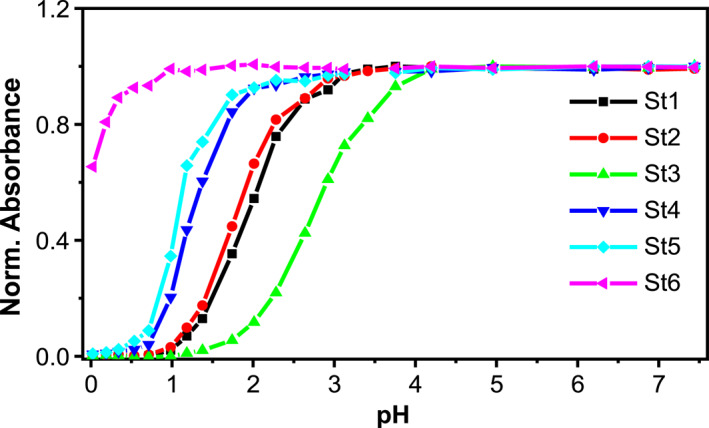
The pH titrations of **St1–6** (5 μM) in an aqueous solution with 0.1% DMSO.

The pH titrations of **St1–6** (5 μM) were then carried out in H_2_O with 0.1% DMSO, pH 7. The absorption spectra at different pH were recorded. By plotting the maximum absorbance intensity against the solution pH, the pH titration curves of compound **P1–7** were established (Figure 3). From the pH titration curves, the acid‐base transitions of **St1**–**5** occurred in the range of 0.5–4, while that of **St6** was predominantly in the negative pH range. By fitting these curves to the Henderson–Hasselbalch equation, their pK_a_ values were calculated to be 1.93, 1.80, 2.76, 1.26, 1.07, and −0.31, respectively. The above spectral studies and pH titrations together confirmed our hypothesis that by replacing the two benzylic carbons of the julolidine, an electron‐donating headgroup was obtained, capable of lowering the pH sensitivity of a fluorophore yet not inducing a hypsochromic shift.

**FIGURE 4:**
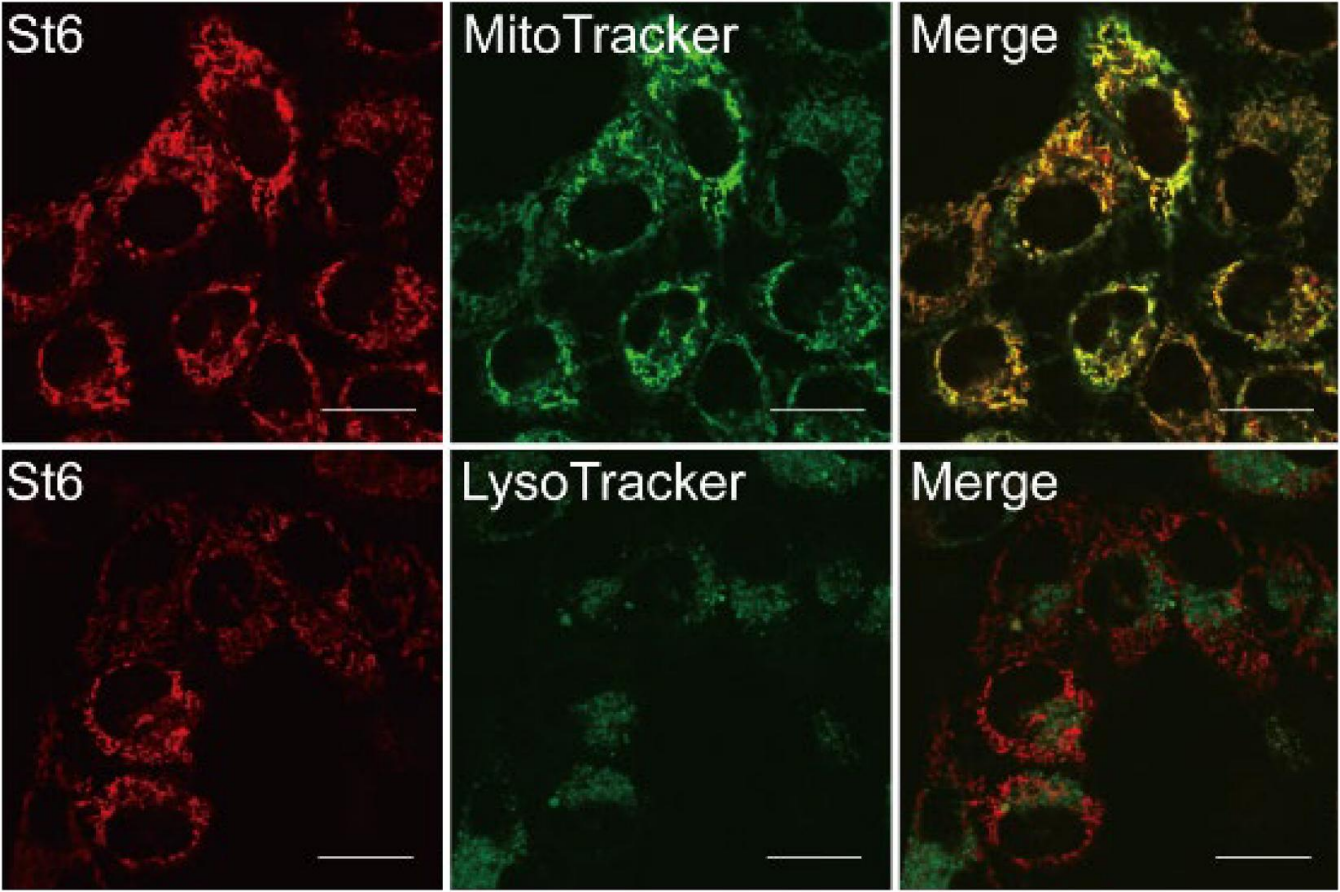
Colocalization fluorescence imaging of Hela cells stained with MitoTracker Green and **St6**, LysoTracker Green and **St6**, respectively. Fluorescence images were collected using confocal microscopy. **St6**: *λ*_ex_ = 541 nm and *λ*_em_ = 629 nm; MitoTracker Green: *λ*_ex_ = 488 nm and *λ*_em_ = 561 nm. LysoTracker Green: *λ*_ex_ = 504 nm and *λ*_em_ = 511 nm. Scale bars: 25 μM.

The potential of **St6** for cell‐imaging studies was checked. The cytotoxicity of **St6** was evaluated in HUVEC and HeLa cells with the CCK8 assay. By incubating with **St6** of varying concentrations (up to 20 μM, respectively) for 24 h, the viability of two cell lines was higher than 80% (Figure S3). This suggested that **St6** was essentially not cytotoxic. **St6** was also permeable to the cell membrane. Incubation for 15 min was sufficient for subsequent cell imaging. The cell localization of **St6** was also investigated with commercial stains for organelles and nuclei. After incubation with **St6** (7.5 μM), cells were treated with MitoTracker Green (100 nM), LysoTracker Green DND‐26 (50 nM), or Hoechst (25 μg/mL), respectively, and the fluorescence images were acquired with a confocal microscope. As shown in Figure 4, the green fluorescence of MitoTracker Green and the red fluorescence of **St6** in HUVEC and Hela cells yielded a high Pearson's correlation coefficient. However, the colocalization of LysoTracker Green and **St6** was not found (Figure S4). These results suggested that **St6** exhibited good cell compatibility and was mitochondria‐targeting.

**FIGURE 5:**
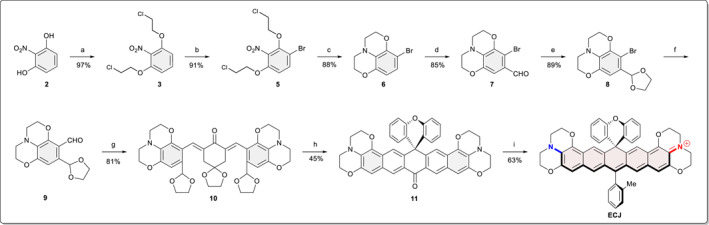
The synthesis of **ECJ**. Reagents and conditions: (a) 1‐bromo‐2‐chloroethane, K_2_CO_3_, CH_3_CN, 90°C, 48 h, 97%; (b) Br_2_, EtOAc, rt, 8 h, 91%; (c) Fe, AcOH, rt −80°C, 6 h, 89%; (d) POCl_3_, DMF, DCE, reflux, 8 h, 85%; (e) ethylene glycol, triethyl orthoformate, TsOH, Toluene, 110°C, 24 h; (f) n‐BuLi, DMF, THF, −78°C, 89%; (g) 1,4‐dioxaspiro[4.5]decan‐8‐one, 30% NaOH, EtOH, rt, 81%; (h) mono‐lithiated diphenyl ether, THF, CH_3_SO_3_H, 0°C; and (i) 2‐methylphenyl lithium, THF, CH_3_SO_3_H, 0°C, 63%.

Based on our previous success with bisbenzannulated carbon‐rhodamine (**ECX**), we further designed and synthesized a NIR fluorophore (**ECJ**) with the headgroup of **D6** (Figure 5). The synthesis of **ECJ** dyes necessitated a 9‐step cascade starting from alkylation of 2‐nitroresocinal (**2**) with 1‐Bromo‐2‐Chloroethane. Electrophilic aromatic substitution of **3** preferentially occurred from *ortho* to the alkoxyl to produce **5** in a 91% yield. Reduction of the nitro group of **6** to amino was achieved with Fe/AcOH by stirring at room temperature. Upon completion of the reduction, the reaction mixture was heated to 90°C for 2 h to promote the intramolecular S_N_2 reaction to prepare **6** in an overall 88% yield. Subsequently, a formyl group was selectively introduced *para* to the nitrogen atom via the Vilsmeier–Haack reaction to yield **7**. The formyl group of **7** was protected by acid‐catalyzed condensation with ethylene glycol to **8**, which was used without further purification. The bromine atom of **8** was lithiated with nBuLi and quenched with anhydrous DMF to give aldehyde **9**, which was then reacted with 1,4‐dioxaspiro[4.5]decan‐8‐one through the base‐catalyzed Aldol condensation reaction to prepare compound **10** in an 81% yield. The carbonyl group of **10** was further added with mono‐lithiated diphenyl ether to yield a carbinol intermediate, the crude residue of which was treated with MeSO_3_H to furnish the diarylketone **11** without purification. **ECJ** was finally synthesized by reacting **11** with 2‐methylphenyl lithium reagent followed by an acid workup in a 63% yield, and the total yield of ECJ was 9.6%.

**FIGURE 6:**
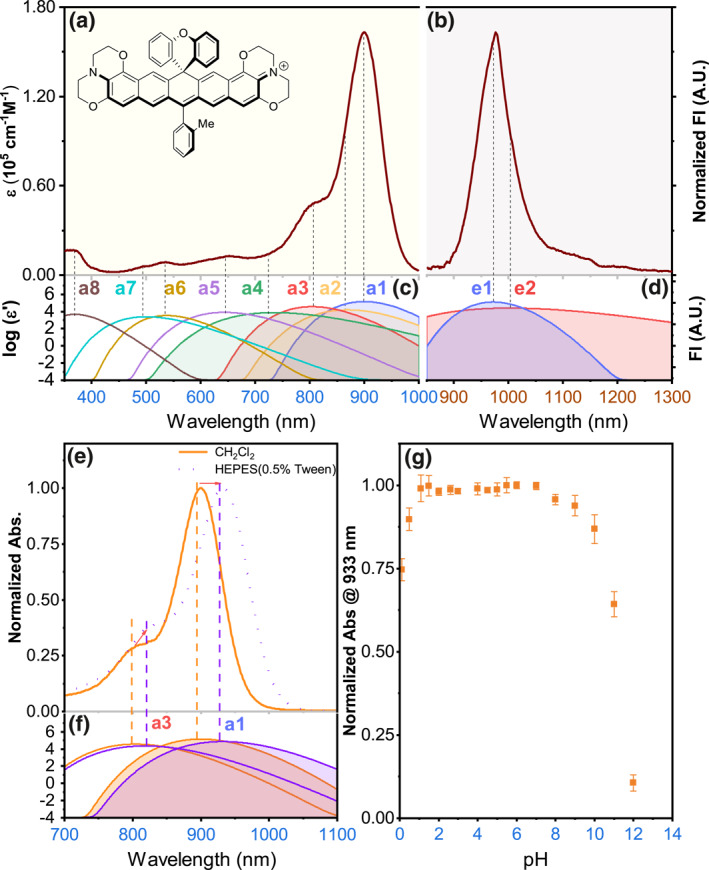
(a, b) The UV‐Vis absorption, the fluorescence emission spectra of **ECJ**. Chemical structures of **ECJ** is included as insets. Absorption and emission spectra are further deconvoluted and the constituting bands are plotted in for (c, d) **ECJ**. (e, f) The absorption spectra and deconvoluted constituting bands of **ECJ** in CH_2_Cl_2_ and HEPES buffer solution (10 mM, pH = 7.4, with 0.5% Tween‐80). (g) The changes of the maximum absorbance of **ECJ** in H_2_O (containing 0.5% Tween‐80% and 0.1% DMSO). The pH was adjusted by adding an aliquot of 1 M HCl solution or 1 M NaOH solution.

The spectral properties of **ECJ** were studied. Its maximal absorption and emission wavelengths were at 901 and 976 nm, respectively (Figure 6a,b, Table 1). Interestingly, these values were longer than those of **ECX**, by 21 and 56 nm, respectively. Its molar absorptivity of *ε* = 1.63 × 10^5^ cm^−1^ M^−1^ was still fairly high, and yet fluorescence quantum yield *ϕ* = 1.2% was considerably lower than **ECX**, typical of a dye with a large Stokes shift. The absorption spectrum of **ECJ** in CH_2_Cl_2_ exhibited a typical cyanine‐type feature, that is, an intense and sharp long‐wavelength band with a shoulder at the short‐wavelength side. The spectrum of **ECJ** in CH_2_Cl_2_ was fitted to a mathematical model of eight constituting Gaussian peaks, that is, *a1*, *a2*, *a3*, *a4*, *a5*, *a6, a7,* and *a8* of decreasing maximal wavelength (Figure 6c,d).[^35^, ^36^] The peaks *a1*–*a4* in the longer spectral region (700–900 nm) are presumably of the HOMO‐LUMO transition from the lowest vibrational level of **S**_**0**_ to the higher vibrational level of **S**_**1**_.[^37^, ^38^] The peaks *a5*–*a8* were presumably vertical excitation to higher excited states. When moved to CH_3_CN, its spectral wavelengths were essentially unchanged, with a decrease in the molar absorptivity and the quantum yield. Its absorption and emission wavelengths exhibited a bathochromic shift in DMSO and HEPES with Tween‐80 by *ca*. 20 nm. The Cyanine spectral feature of the **ECJ** was maintained in those polar solvents (Figure 6e,f).

**TABLE 1:** The photophysical parameters of **ECJ**.

Solvent	*λ*_abs_/nm	*λ*_em_/nm	*ε*/cm^−1^ • M^−1^	*ϕ*f^a^
CH_2_Cl_2_	901	976	1.63 × 10^5^	0.012
MeCN	902	980	1.13 × 10^5^	0.009
DMSO	927	1008	8.90 × 10^4^	0.010
HEPES^b^	933	1003	1.02 × 10^5^	0.005

^a^ The fluorescence quantum yield determined using **ECXb** as reference (*ϕ* = 0.111 in chloroform).

^b^ HEPES buffer solution (pH = 7.4, 10 mM, containing 0.5% Tween‐80% and 0.1% DMSO).

**FIGURE 7:**
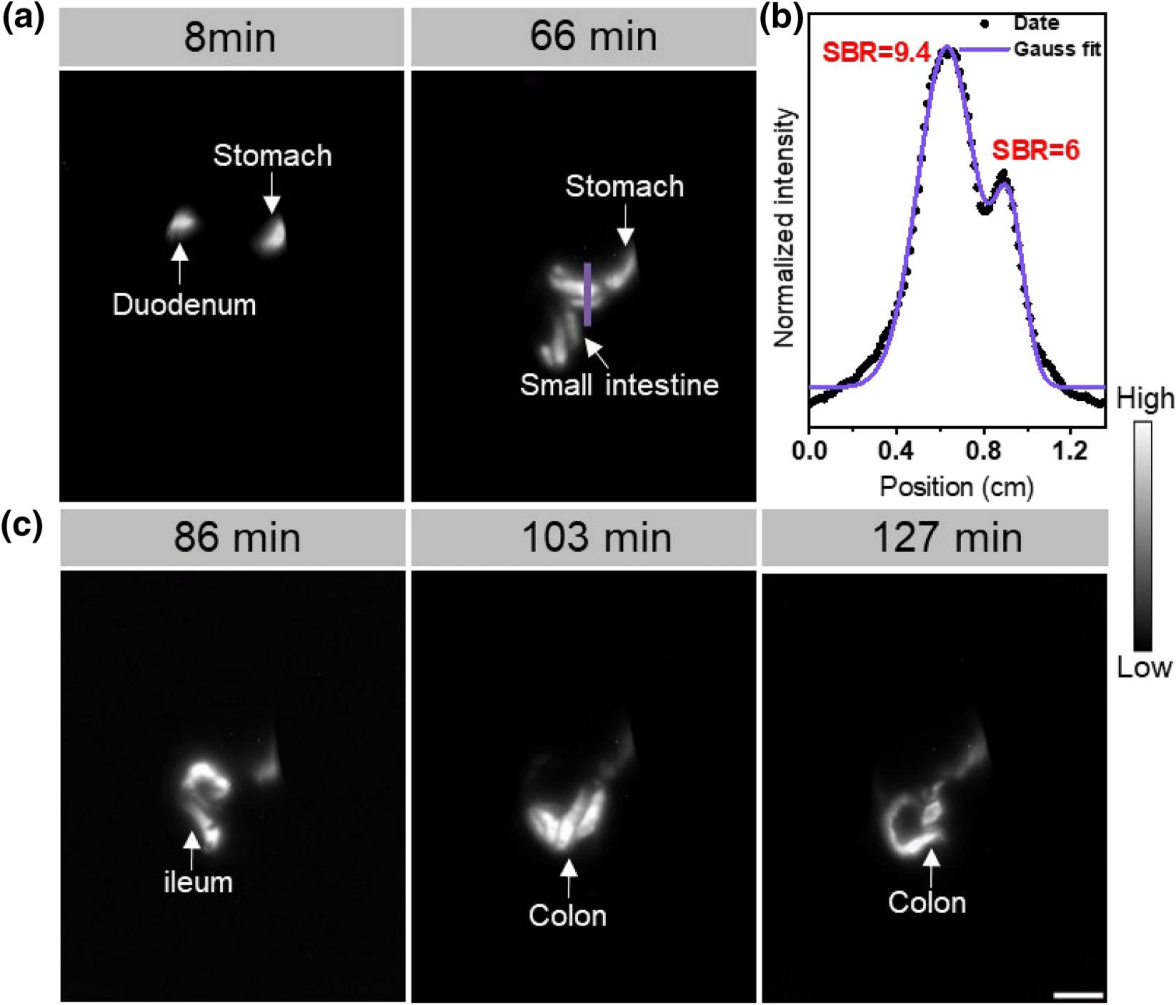
In vivo gastrointestinal tract fluorescence imaging of nude mice. (a, c) Gastrointestinal tract bioimaging after intragastric injection of **ECJ** (0.4 mg/kg, PBS containing 1% Tween‐80% and 0.1% DMSO) with 980 nm laser line (1150 nm longpass filter, 20 ms, 150 mW/cm^2^) at different time points, scale bar: 1 cm. (b) The signal‐to‐background ratio and the calculated diameter of the selected intestines in (c) at 66 min.

We then performed a proof‐of‐concept in vivo imaging with **ECJ**. With the acid resistance of **ECJ**, it could potentially be used to image the gastrointestinal tract, particularly the stomach. The pH of the human gastric acid is low at ca. 1. The pH of mouse gastric acid is slightly higher at 2–3, which is still challenging for many other long‐wavelength dyes, including **ECX**. A BALB/c mouse was intragastrically administrated **ECJ** (0.4 mg/kg) and a fluorescence image was first acquired in 66 min with a 930 nm laser excitation (Figure 7b). The emission beyond 1150 nm was collected with a longpass and the exposure time was 20 ms. Three additional images were acquired at the time points of 86, 103, and 127 min; the whole process of the excretion of **ECJ** along the intestine tract was clearly delineated. At the time point of 86 min, the fluorescence signal of **ECJ** was observed from both the stomach and the intestine. A high signal‐to‐background contrast of 9.4 was calculated. The diameters of the two sectors of intestinal tracts were calculated to be 3.1 and 1.8 mm, respectively (Figure 7c). With the last image of 127 min, **ECJ** went further down to highlight a different sector of the intestine tract. A residual signal was still apparent in the stomach. With this experiment, we showcased that **ECJ** could indeed be used to highlight the acidic medium for in vivo bioimaging.

## 3 CONCLUSION

By rational design, we proposed a new electron‐donating group (**D6**) for the construction of an NIR fluorophore (**ECJ**), whose spectral properties are unaffected even in a highly acidic medium. They have the potential for bioimaging of the gastrointestinal tract. We got the inspiration from the lower pK_a_ of the nitrogen atoms of a julolidine or a morpholine. By combining their effects, we devised **D6**. With a styrylindolium fluorochromic scaffold, we verified that the analog (**St6**) with **D6** indeed imparted a lower pK_a_ than ones with conventional electron‐donating headgroups. We further developed a synthesis of **ECJ**, whose absorption and emission wavelengths were located in the deep NIR spectral region beyond 900 nm. Its feasibility for in vivo imaging in gastric acid was showcased with a mouse model.

## CONFLICT OF INTEREST STATEMENT

The authors declare no conflict of interests.

## ETHICS STATEMENT

All animal experiments were performed according to the guidelines of the Care and Use of Laboratory Animals formulated by the Ministry of Science and Technology of China and were approved by the Animal Care and Use Committee of East China Normal University.

## Supporting information

Supporting Information S1

## Data Availability

The data that support the findings of this study are available from the corresponding authors upon reasonable request.
