# Supplementary material for: An acidic medium‐compatible deep‐near‐infrared dye for in vivo imaging
Source: Smart Mol. 2023 May 16;1(1):e20230001. doi: 10.1002/smo.20230001 (PMC12118197; doi:10.1002/smo.20230001)
Supplement: Supplementary file 1 — Supporting Information S1 [file SMO2-1-e20230001-s001.docx]

**Supporting information**

Acidic Medium-Compatible Deep-NIR-Infrared Dye for In vivo Imaging

Yan Dong^a^, Ye Zou^a^, Xiaotong Jia^a^, Lei Yin^a^, Weiwei He^a^, Xiao Luo^b^, Xuhong Qian^b^, Youjun Yang^a,^ ^^[[1]](#footnote-1)^*^

*^a^ State Key Laboratory of Bioreactor Engineering, School of Pharmacy, East China University of Science and Technology, Shanghai 200237, China*

*^b^ School of Chemistry and Molecular Engineering, East China Normal University, Shanghai 200062, China*

Table of Contents

| Page | Content | |
| --- | --- | --- |
| S3-S4 |  | General methods. |
| S5 | Figure S1 | Photo-stability studies of **ECJ** and **ICG**. |
| S5 | Figure S2-3 | Cell viability test of **ECJ** and **St1-6**. |
| S5 | Figure S4 | Pearson's correlation coefficient studies of **St6**. |
| S6 | Tables S1 | Photophysical property of **St1-6**. |
| S7-S9 |  | Synthesis and compound characterizations. |
| S10-S38 |  | The ^1^H-/^13^C-NMR and HRMS spectra of all compounds. |

General methods

All chemical reagents were analytically pure and purchased from chemical sources, e.g., Aladdin Reagent, TCI Shanghai, Sinopharm Chemical Reagent, Energy Chemical, Adamas-beta based in China and used as received. Deionized water was obtained from Millipore Milli-Q SP water purification system. The organic solvent used in the purification of compounds by column chromatography were purchased from Titan scientific, e.g. dichloromethane (DCM), methanol, ethanol, petroleum ether (PE), ethyl acetate (EtOAc), acetonitrile (MeCN), dimethyl sulfoxide (DMSO) was bought from Adamas-beta with activated 4A molecular sieves. Anhydrous tetrahydrofuran (THF) was dried over sodium benzophenone still.

Instruments

All the ^1^H-NMR and ^13^C-NMR spectra were measured on Bruker AV-400 or AV-600 spectrometers. Chemicals shifts are referenced to the residue solvent peaks and given in ppm. HRMS were acquired on a Micromass GCT spectrometer.

Absorbance spectra were performed on a SHIMADZU UV-2600 UV-vis spectrometer. The Fluorescence emission spectra were recorded on a PTI-QM4 steady-stead fluorimeter with a 75 W Xe lamp, a R928 PMT and an InGaAs photodetector, quartz cuvette (1 cm) was used for measurements.

The NIR-II *in vivo* fluorescence imaging was performed by a small animal imaging system (MARS, Artemis Intelligent Imaging, Shanghai, China). The excitation illumination was provided by a 980 nm laser device equipped with a shadowless equipment. The emitted light was collimated by a 50 mm focal length SWIR lens (MARS-FAST, Artemis Intelligent Imaging) after passing through long-pass filters. The fluorescence images were captured by an electronic-cooling InGaAs camera (NIRvana, Teledyne Princeton Instruments).

Fluorescence quantum yield determination

The relative fluorescence quantum yields of **ECJ** in different solvents were measured with **ECXb** in CHCl_3_ as the reference.^1^ A detailed protocol for determination of the relative fluorescence quantum yield is provided below.^2^

1. Lei, Z.; Li, X.; Luo, X.; He, H.; Zheng, J.; Qian, X.; Yang, Y. *Angew. Chem. Int. Ed*., **2017**, *56*, 2979-2983.
2. Würth, C.; Grabolle, M.; Pauli, J.; Spieles, M.; Resch-Genger, U. *Nat. Protocols*, **2013**, *8*, 1535–1550.

Photo-stability studies

The photostability of **ECJ** and **ICG** were studied with a home-built NIR epi-fluorescence microscope with an 808 nm laser. A solution of these dyes with an absorbance of 0.25 at 808 nm was dropped onto a slide and sealed with coverslip, then the slides were illuminated with a power density of 3.16 W/cm^2^. The fluorescence images of these dyes on the slide were acquired during the time of illuminating.

Animal handling

All the BALB/c mice were purchased from Shanghai JieSiJie Laboratory Animals. All the animal experiments were performed strictly following protocols approved by the Institutional Animal Care and Use Committee (IACUC) of the Shanghai Institute of Material Medica, Chinese Academy of Sciences. Mice were selected randomly from cages for all the imaging studies. Six-week-old female BALB/c mice were used for *in vivo* imaging studies. Before imaging, the mice were anaesthetized with air mixed with 3% isoflurane. During the time course of imaging, the mouse was kept anaesthetized by mask. All long-pass filters were from Thorlabs. All the images were processed using the Fiji distribution of ImageJ.

***In vivo* gastrointestinal (GI) tract fluorescent imaging**

BALB/c mice, weight ~20 g, were used for *in vivo* whole-body and gastrointestinal tract dual-color NIR-II fluorescence imaging. After intragastric injection of **ECJ** (0.4 mg/kg, PBS containing 0.5% Tween-80 and 0.1% DMSO), the mice were permitted to move freely for 1 hr. Imaging the mouse in a supine position with the excitation of 980 nm (1150 nm longpass filter, 20 ms exposure time, 150 mW/cm^2^) laser line at different time points. All the images were collected on the MARS imaging system.

**Cell viability studies**

The cytotoxicity was measured by using Cell Counting Kit-8(CCK-8) assay. Hela cells(1Х10^4^) were plated into a 96-well plate for 24 h in Dulbecco’s modified Eagle medium (DMEM) with 10% FBS at 37 °C and 5% CO_2_. Then, the cells were incubated with **ECJ** at different concentrations (10 μM, 8 μM, 6 μM, 4 μM, 2 μM, 0 μM) for 24 h, respectively. Besides, the cells were also incubated with **St6** at different concentrations (20 μM, 10 μM, 7.5 μM, 5 μM, 2.5 μM, 0 μM) for 24 h, respectively. 24 h later, 10 μL CCK-8 was added into each well for additional 40 min incubation. The absorbance was assessed with a microplate reader (Biotec-SynergyTM NEO) at a wavelength of 450 nm. Following formula was used to calculate the viability of cell growth:

Viability (%) = (mean absorbance value of treatment group/mean absorbance value of control group) Х 100.**Photo-stability tests**

**Figure S1.** Photostability studies of **ECJ** and **ICG**.

**Cell viability**

**
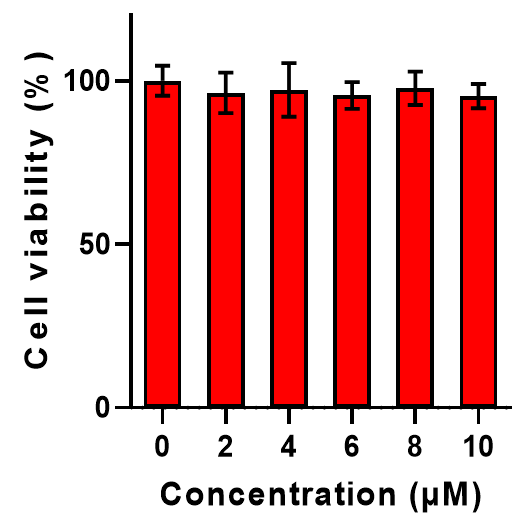
**

**Figure S2.** Cell viability of Hela treated with **ECJ** at various concentration ranging from 0 to 10 μM for 24 h. Date are presented as mean ± s.d. derived from n = 6 independent measurements (Unqualified date were excluded).

**Figure S3.** Cell viability of Hela treated with **St6** at various concentration ranging from 0 to 20 μM for 24 h. Date are presented as mean ± s.d. derived from n = 6 independent measurements (Unqualified date were excluded).


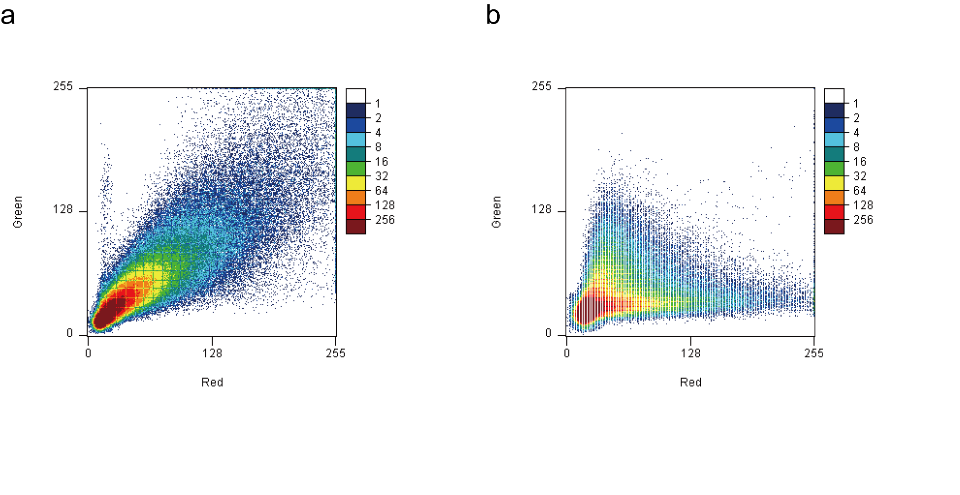


**Figure S4.** Scatter plot of green and red images for the calculation of Pearson's correlation coefficient. (a) MitoTracker Green and **St6**. (b) LysoTracker Green and **St6**.

**Table S1.** The photophysical parameters of **St1-6**.

| Compound | λ_abs_/nm | λ_em_/nm | Stokes shift/nm | ε/cm^-1^•M^-1^ | φ^* ^ |
| --- | --- | --- | --- | --- | --- |
| **St1** | 508 | 555 | 47 | 0.69 × 10^5^ | 0.37 |
| **St2** | 563 | 595 | 32 | 1.80× 10^5^ | 0.24 |
| **St3** | 570 | 599 | 29 | 1.85× 10^5^ | 0.27 |
| **St4** | 554 | 596 | 42 | 0.96× 10^5^ | 0.32 |
| **St5** | 592 | 619 | 27 | 2.31 × 10^5^ | 0.06 |
| **St6** | 583 | 625 | 42 | 1.16 × 10^5^ | 0.16 |

Note: ^*^ fluorescence quantum yield determined using **Rhodamine B** as reference (φ = 0.31 in H_2_O)

**Synthesis and compound characterizations**

**General synthesis procedure for 2-styrylindolium derivatives (St1-6)**: A mixture of 1,2,3,3-Tetramethyl-3H-indolium iodide (200 mg, 1.49 mmol, 0.9 eq) and the corresponding aldehyde (1.65 mmol, 1 eq) was dissolved in EtOH (20 mL). The mixture was then stirred at 70 °C for 8 h under nitrogen. The solvent was evaporated *in vacuo*. The crude product was purified by a flash chromatography (silica, CH_2_Cl_2_: MeOH=10:1) to give 2-styrylindolium derivatives as solid. The ^1^H-NMR spectra of compound **St1-5** were same as the previous reports.

1. J. Gu, U. Anumala, F. Monte, T. Kramer, R. Hauben, J. Holzer, V. Meyer, G. Mall, I. Hilger, C. Czech, B. Schmidt. *Bioorg. Med. Chem. Lett*., 2012, **22**, 7667-7671.

2. S. Metsov, D. Simov, S. Stoyanovb, P. Nikolov. Dyes Pigm, 1990, **13**, 11-19.

Synthesis of **St1**. Prepared from 4-aminobenzaldehyde as a dark pink solid (450 mg, 75%); HRMS (ES^+^) m/z calcd for C_19_H_21_N_2_^+^[M]^+^, 277.1699; Found, 277.1706.

Synthesis of **St2**. Prepared from 4-(dimethylamino)benzaldehyde as a dark green solid (455 mg, 87%); HRMS (ES^+^) m/z calcd for C_21_H_25_N_2_^+^[M]^+^, 305.2012; Found, 305.2017.

Synthesis of **St3**. Prepared from 4-(diethylamino)benzaldehyde as a dark green solid (387 mg, 84%); HRMS (ES^+^) m/z calcd for C_23_H_29_N_2_^+^[M]^+^, 333.2325; Found, 333.2330.

Synthesis of **St4**. Prepared from 4-morpholinobenzaldehyde as a dark green solid (345 mg, 77%); HRMS (ES^+^) m/z calcd for C_23_H_27_N_2_O^+^[M]^+^, 347.2118; Found, 347.2122.

Synthesis of **St5**. Prepared from 2,3,6,7-tetrahydro-1H,5H-pyrido[3,2,1-ij]quinoline-9-carbaldehyde as a dark green solid (345 mg, 80%); HRMS (ES^+^) m/z calcd for C_25_H_29_N_2_^+^[M]^+^, 357.2325; Found, 357.2332.

Synthesis of **St6**. Prepared from 2,3,4,5-tetrahydro-1,6-dioxa-3a-azaphenalene-8-carbaldehyde as a dark green solid (310 mg, 72%); ^1^H NMR (600 MHz, CDCl_3_) δ 7.94 (d, *J =* 15.4 Hz, 1H), 7.58 (d, *J =* 8.0 Hz, 1H), 7.54-7.51 (m, 1H), 7.50-7.43 (m, 2H), 7.30 – 7.25 (d, 1H), 7.16 (s, 2H), 4.35 (dd, *J* = 5.1, 3.8 Hz, 4H), 4.24 (s, 3H), 3.54 (t, *J* = 4.5 Hz, 4H), 1.77 (s, 6H); ^13^C NMR (151 MHz, CDCl_3_) δ 179.34, 154.91, 143.39, 142.02, 141.91, 131.25, 129.48, 128.32, 122.90, 122.29, 113.76, 113.13, 106.65, 63.93, 51.26, 46.62, 34.94, 27.41; HRMS (ES^+^) m/z calcd for C_23_H_25_N_2_O_2_^+^[M]^+^, 361.1911; Found, 361.1915.

Synthesis of **1,3-bis(2-chloroethoxy)-2-nitrobenzene** (**3**)**.** 2-nitrobenzene-1,3-diol (20 g, 129 mmol, 1equiv.), 1-bromo-2-chloroethane (54 ml, 645 mmol, 5equiv.), K_2_CO_3_ and MeCN (200 ml) were mixed in a round bottom flask and heated in an oil-bath at 90 °C for 48 hours with rigorous stirring. The reaction mixture was cooled to room temperature and filtered. The filtrate was then poured into petroleum ether. The solid was filtered and dried to give **3** (35 g, 97%) as a white powder. ^1^H NMR (400 MHz, CDCl_3_) δ 7.34 (t, *J =* 8.0 Hz, 1H), 6.67 (d, *J =* 8.0 Hz, 2H), 4.30 (t, *J =* 4.0 Hz, 4H), 3.78 (t, *J =* 4.0 Hz, 4H); ^13^C NMR (101 MHz, CDCl_3_) δ 150.5, 132.8, 131.3, 106.5, 69.5, 41.0; HRMS (ES^+^) calcd for C_10_H_11_Cl_2_NO_4_ [M+Na]^+^, 301.9957; Found, 301.9961.

Synthesis of **1-bromo-2,4-bis(2-chloroethoxy)-3-nitrobenzene** (**5**)**.** The compound **3** (10 g, 35.7 mmol, 1equiv.), Bromine (2.74 ml, 1.5equiv.), and ethyl acetate (200 ml) were charged in a 500 ml round bottom flask and stirred at room temperature overnight. The reaction was quenched by addition of aqueous NaOH and extracted with EtOAc (3 × 100 mL). The organic layer was dried over anhydrous Na_2_SO_4_, then filtered and evaporated to dryness to give **5** (11.7 g, 91%) as a yellow oil with no further purification. ^1^H NMR (400 MHz, CDCl_3_) δ 7.60 (d, *J =* 12.0 Hz, 1H), 6.76 (d, *J =* 8.0 Hz, 1H), 4.36 (t, *J =* 8.0 Hz, 2H), 4.30 (t, *J =* 8.0 Hz, 2H), 3.79 (t, *J =* 8.0 Hz, 2H), 3.78 (t, *J =* 8.0 Hz, 2H); ^13^C NMR (101 MHz, CDCl_3_) δ 149.7, 148.1, 137.9, 134.8, 111.0, 108.8, 74.4, 69.9, 41.6, 40.9; HRMS (ES^+^) calcd for C_10_H_10_BrCl_2_NO_4_ [M+Na]^+^, 379.9062; Found, 379.9070.

Synthesis of **9-bromo-2,3,4,5-tetrahydro-1,6-dioxa-3a-azaphenalene** (**6**)**.** The compound **5** (20 g, 55.75 mmol, 1equiv.), ethanol (100 ml), acetic acid (2.2 ml, 39 mmol, 0.7 equiv.) and ferrous powder (12.45 g, 223 mmol, 4equiv.) were added into a 250 ml round bottom flask and the mixture was heated to 90 °C for 12 hours under Ar atmosphere. The reaction mixture was filtered and the filtrate was adjusted to pH=12 by addition of aqueous NaOH. Then the mixture was extracted with CH_2_Cl_2_ (3 × 100 mL) and H_2_O. The organic layer was dried over anhydrous Na_2_SO_4_, filtered and evaporated to dryness. The crude product was purified by a flash chromatography [silica, Petroleum ether:CH_2_Cl_2_, 1/1] to give **6** (12.4 g, 87%) as a bluish violet solid. ^1^H NMR (400 MHz, CDCl_3_) δ 6.80 (d, *J =* 8.0 Hz, 1H), 6.31 (d, *J =* 8.0 Hz, 1H), 4.47 (t, *J =* 4.0 Hz, 2H), 4.36 (t, *J =* 4.0 Hz, 2H), 3.18 (t, *J =* 4.0 Hz, 2H), 3.17 (m, 2H); ^13^C NMR (101 MHz, CDCl_3_) δ 143.6, 140.8, 122.9, 121.8, 109.2, 100.6, 65.7, 65.0, 46.5, 46.4; HRMS (ES^+^) calcd for C_10_H_10_BrNO_2_ [M+H]^+^, 255.9968; Found, 255.9970.

Synthesis of **9-bromo-2,3,4,5-tetrahydro-1,6-dioxa-3a-azaphenalene-8-carbaldehyde** (**7**)**.** POCl_3_ (21.8 ml, 234.3 mmol, 2 equiv.) and DMF (150 ml) were mixed and stirred for 40 min at ice-bath and then transferred into a solution of compound **6** (30 g, 117.1 mmol, 1 equiv.) in 1,2-Dichloroethane. The mixture was heated to 80 °C for 6 hours before cooled to r.t. and then poured into ice water. The brown solid was filtered and dried to give **7** (28.3 g, 85%) without further purification. ^1^H NMR (400 MHz, CDCl_3_) δ 10.16 (s, 1H), 7.10 (s, 1H), 4.48 – 4.42 (t, *J =* 4.0 Hz, 2H), 4.36 – 4.30 (t, *J =* 4.0 Hz, 2H), 3.34 (m, 4H); ^13^C NMR (151 MHz, CDCl_3_) δ 196.0, 142.4, 139.4, 128.8, 123.1, 109.8, 107.9, 64.8, 64.1, 46.1, 46.0; HRMS (ES^+^) calcd for C_11_H_10_BrNO [M+H]^+^, 283.9917; Found, 283.9925.

Synthesis of **9-bromo-8-(1,3-dioxolan-2-yl)-2,3,4,5-tetrahydro-1,6-dioxa-3a-azaphenalene** (**8**). The compound **7** (10 g, 35.2 mmol, 1 equiv.), ethylene glycol (3.28 g, 52.8 mmol, 1.5 equiv.) and triethyl orthoformate (5.22 g, 35.2 mmol, 1 equiv.) were dissolved in toluene (200 ml) before heated to 100 °C, then p-toluenesulfonic acid (0.6 g, 3.52 mmol, 0.1 equiv.) was added and stirred at 110 °C overnight. The mixture was cooled to r.t. and quenched with aqueous sodium hydrogen carbonate. The crude product was extracted with CH_2_Cl_2_ and purified by a flash column [silica, PE/EA/Et_3_N 100/10/1] to give **8** (8.4 g, 73%) as a pale yellow solid. ^1^H NMR (400 MHz, CDCl_3_) δ 6.68 (s, 1H), 6.03 (s, 1H), 4.48 – 4.40 (m, 2H), 4.34 – 4.28 (m, 2H), 4.12 – 4.04 (m, 2H), 4.03 – 3.96 (m, 2H), 3.20 – 3.12 (m, 4H).

Synthesis of **8-(1,3-dioxolan-2-yl)-2,3,4,5-tetrahydro-1,6-dioxa-3a-azaphenalene-9-carbaldehyde** (**9**). A solution of **nBuLi** (14.6 ml, 45.7 mmol, 1.2 equiv.) was added into a solution of compound **8** (10 g, 30.5 mmol, 1 equiv.) drop wise in 200 ml anhydrous THF at -78 °C (EtOAc-liquid N2 bath). The mixture was stirred for 20 min before anhydrous DMF (3.54 ml, 45.7 mmol, 1.5 equiv.) was added. The mixture was then allowed to warm to r.t. within two hours and then quenched with aqueous NH_4_Cl. The crude product was extracted with CH_2_Cl_2_ and purified by a flash column [silica, PE/EA/Et_3_N 100/25/1] to give **9** (7.3 g, 89%) as a pale yellow solid. ^1^H NMR (400 MHz, CDCl_3_) δ 10.38 (s, 1H), 6.82 (s, 1H), 6.53 (s, 1H), 4.53 – 4.45 (m, 2H), 4.44 – 4.39 (m, 2H), 4.14 – 3.96 (m, 4H), 3.29 – 3.13 (m, 4H). ^13^C NMR (151 MHz, CDCl_3_) δ 189.7, 149.1, 148.4, 131.0, 115.8, 107.2, 99.9, 65.6, 65.2, 46.4; 46.0; HRMS (ES^+^) calcd for C_14_H_15_NO_5_ [M+Na]^+^, 300.0842; Found, 300.0853.

Synthesis of **(7E,9E)-7,9-bis((8-(1,3-dioxolan-2-yl)-2,3,4,5-tetrahydro-1,6-dioxa-3a-azaphenalen-9-yl)methylene)-1,4-dioxaspiro[4.5]decan-8-one** **(10).** The Compound **9** (10 g, 36 mmol, 2 equiv.) and 1,4-dioxaspro [4,5]decan-8-one (2.82 g, 18 mmol, 1 equiv.) were dissolved in 20 ml EtOH. To this solution, 40% NaOH aq. (1 ml) was added slowly at r.t. with rigorous stirring overnight. The precipitate was filtered and dried to give **10** (9.9 g, 81%) as a yellow solid. ^1^H NMR (600 MHz, CDCl_3_) δ 7.81 (s, 2H), 6.66 (s, 2H), 5.70 (s, 2H), 4.41 – 4.31 (m, 8H), 4.13 – 4.04 (m, 4H), 3.97 – 3.90 (m, 4H), 3.83 (s, 2H), 3.18 – 3.15 (m, 4H), 3.15 – 3.12 (m, 4H), 2.72 (s, 4H). ^13^C NMR (151 MHz, CDCl_3_) δ 186.5, 144.1, 135.8, 134.3, 126.6, 122.5, 115.9, 107.3, 106.7, 65.3, 65.2, 64.4, 46.7; 46.4, 37.4; HRMS (ES^+^) calcd for C_36_H_38_N_2_O_11_ [M+H]^+^, 675.2548; Found, 675.2553.

Synthesis of **11**. A solution of **nBuLi** (4.7 ml, 11.9 mmol, 4 equiv.) was slowly added into the solution of diphenyl ether (2 g, 11.9 mmol, 4 equiv.) in 30 ml anhydrous THF at ice-bath. The resulting mixture was stirred for 40 min before adding to a solution of compound **10** (2 g, 2.9 mmol, 1 equiv.) in anhydrous THF (100 ml) under rigorous stirring. The reaction was monitored by TLC analysis and it was completed in 2 hours. Then the reaction was quenched with aqueous NH_4_Cl. The mixture was extracted with CH_2_Cl_2_ (3 × 50 ml) and the solvent was evaporated *in vacuo*. Then the residue was added CH_2_Cl_2_ (100 ml) and methanesulfonic acid (2 ml). The mixture was stirred for 4 h before worked up with aqueous sodium hydrogen carbonate. The crude product was extracted with CH_2_Cl_2_ and purified by recrystallization [PE/CH_2_Cl_2_ 100/20] to give **11** (0.88 g, 45%) as a brown solid.

Synthesis of **ECJ**. A solution of **nBuLi** (6.1 ml, 15.2 mmol, 10 equiv.) was slowly added into the solution of 1-Bromo-2-methybenzene (2.6 g, 15.2 mmol, 10 equiv.) in 30 ml anhydrous THF at -78 **°**C (EtOAc-liquid N_2_ bath). The resulting mixture was stirred for 20 min and transferred to a solution of compound **11** (1 g, 1.52 mmol, 1 equiv.) in anhydrous THF (150 ml) at 0 **°**C. Into the reaction mixture was added methanesulfonic acid (2 ml). The mixture was stirred for 8 h before worked up with aqueous sodium hydrogen carbonate. The crude product was extracted with CH_2_Cl_2_ and purified by a flash chromatography [silica, CH_2_Cl_2_/MeOH, 20/1] to give **ECJ** (700 mg, 63%) as a bluish violet solid. ^1^H NMR (600 MHz, CDCl_3_) δ 7.60 (t, J = 7.4 Hz, 1H), 7.56 – 7.47 (m, 2H), 7.45 – 7.34 (m, 5H), 7.30 (d, J = 8.2 Hz, 2H), 7.24 (s, 2H), 6.87 (dt, J = 13.9, 7.4 Hz, 2H), 6.78 – 6.63 (m, 4H), 4.33 (m, 8H), 3.73 (m, 8H), 2.24 (s, 2H); ^13^C NMR (151 MHz, CDCl_3_) δ 148.9, 130.7, 129.9, 129.8, 129.7, 128.3, 126.0, 125.0, 123.8, 123.8, 117.3, 117.2, 109.9, 64.1, 63.8, 47.3, 47.2, 31.5, 19.9; HRMS (ES^+^) *m/z* calcd for C_49_H_37_N_2_O_5_ [M]^+^, 733.2697; Found, 733.2701.


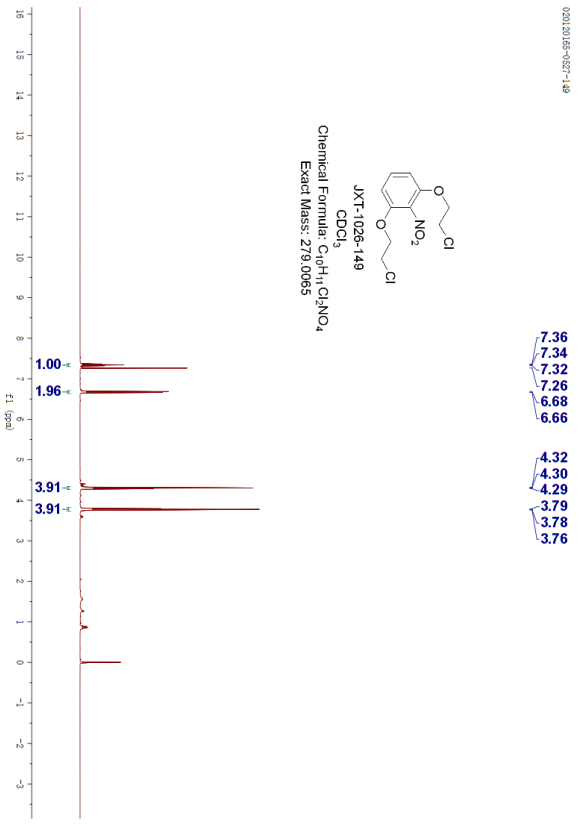


**Figure S5.** The ^1^H-NMR of compound **3** in CDCl_3_.

**_
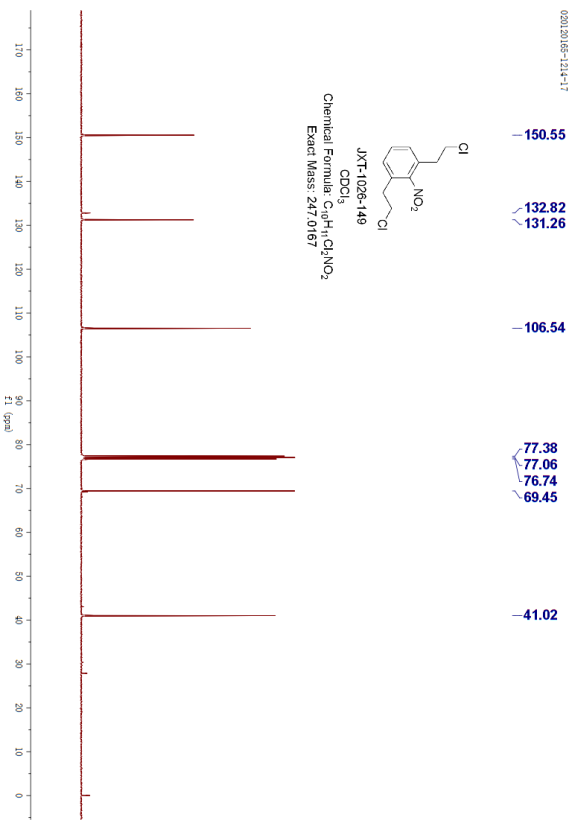
_**

**Figure S6.** The ^13^C-NMR of compound **3** in CDCl_3_.


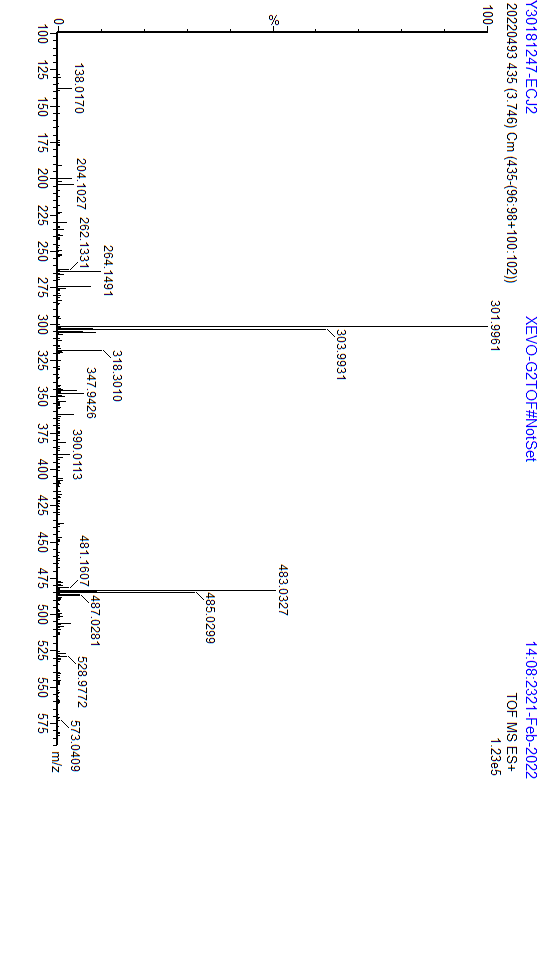


**Figure S7.** The HR-MS of compound **3**.

_
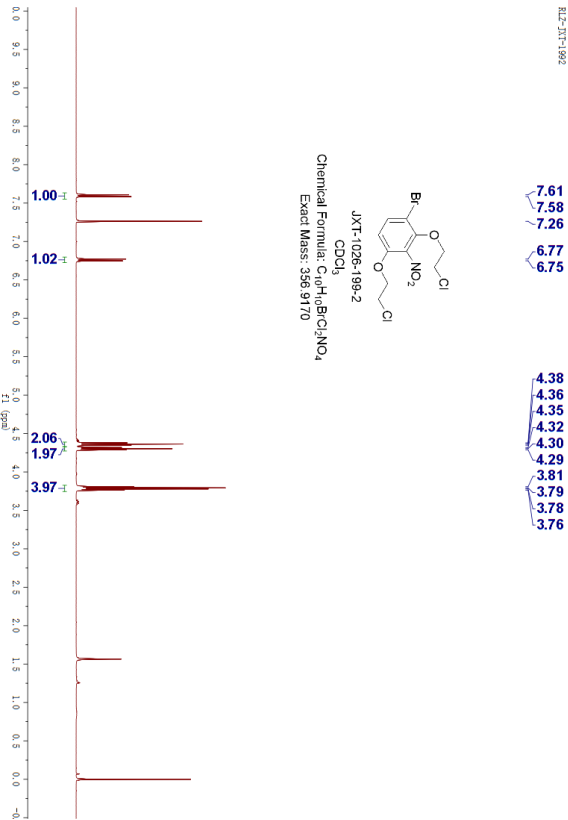
_

**Figure S8.** The ^1^H-NMR of compound **5** in CDCl_3_.


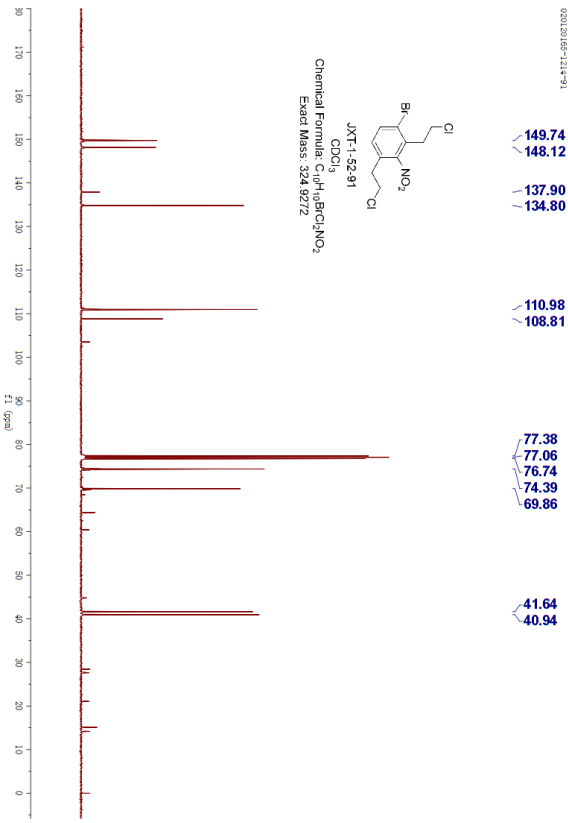


**Figure S9.** The ^13^C-NMR of compound **5** in CDCl_3_.


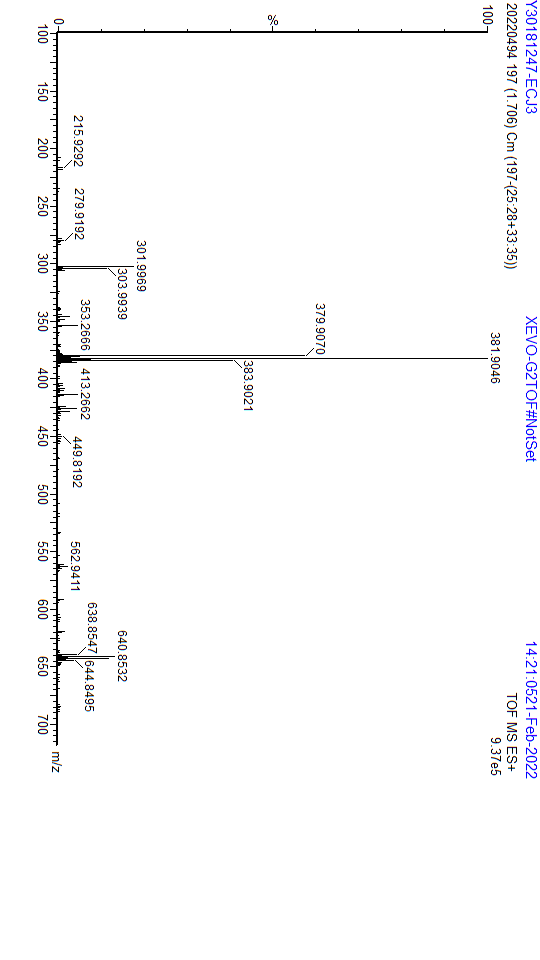


**Figure S10.** The HR-MS of compound **5**.


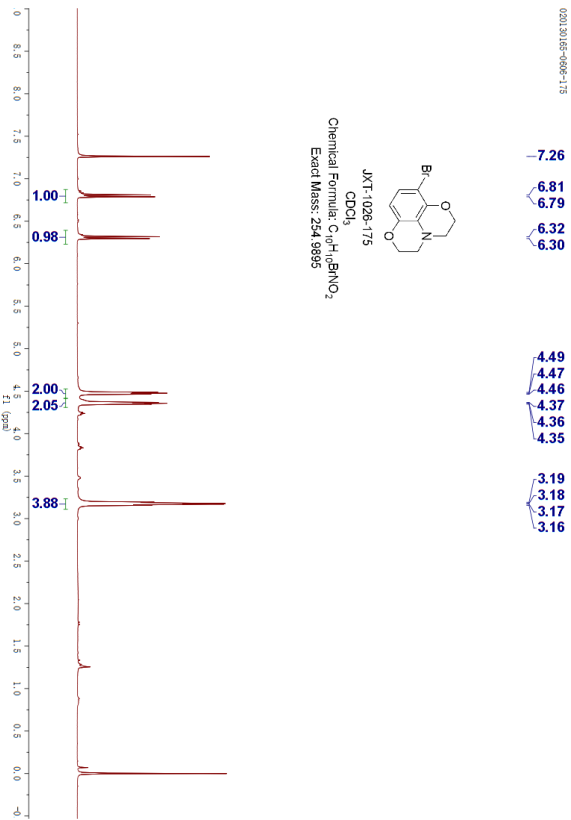


**Figure S11.** The ^1^H-NMR of compound **6** in CDCl_3_.


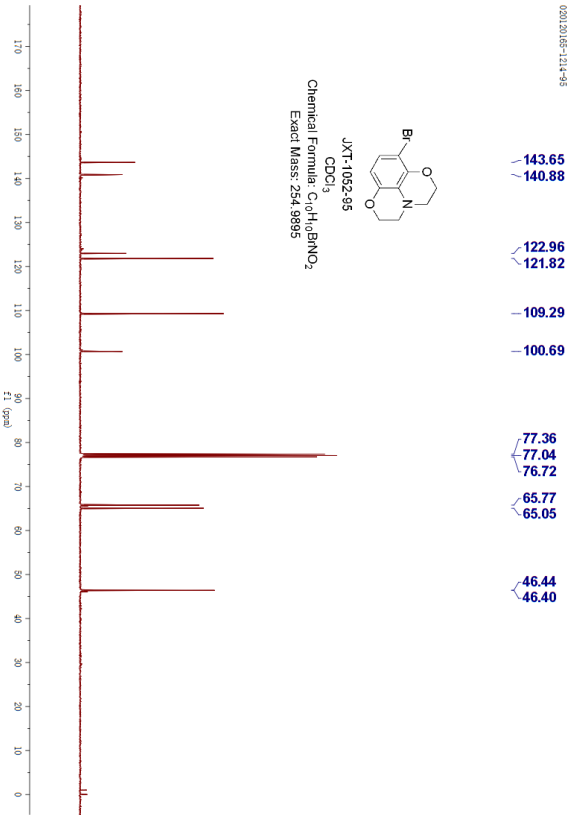


**Figure S12.** The ^13^C-NMR of compound **6** in CDCl_3_.


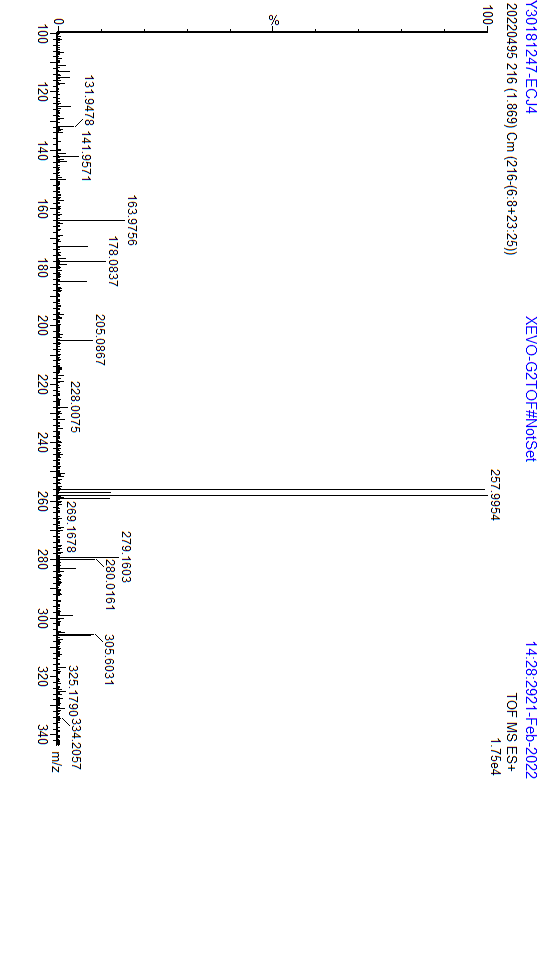


**Figure S13.** The HR-MS of compound **6**.


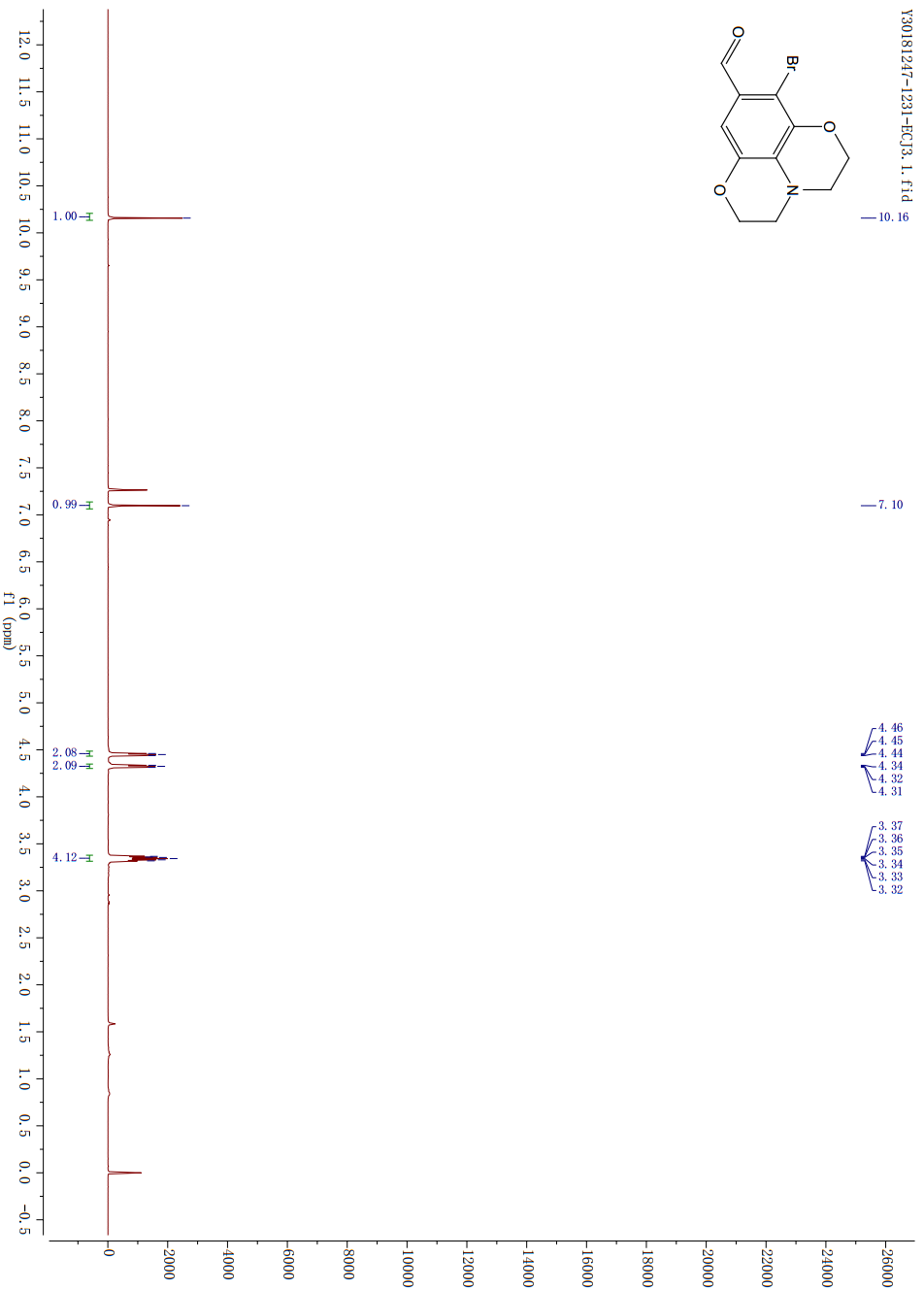


**Figure S14.** The ^1^H-NMR of compound **7** in CDCl_3_.


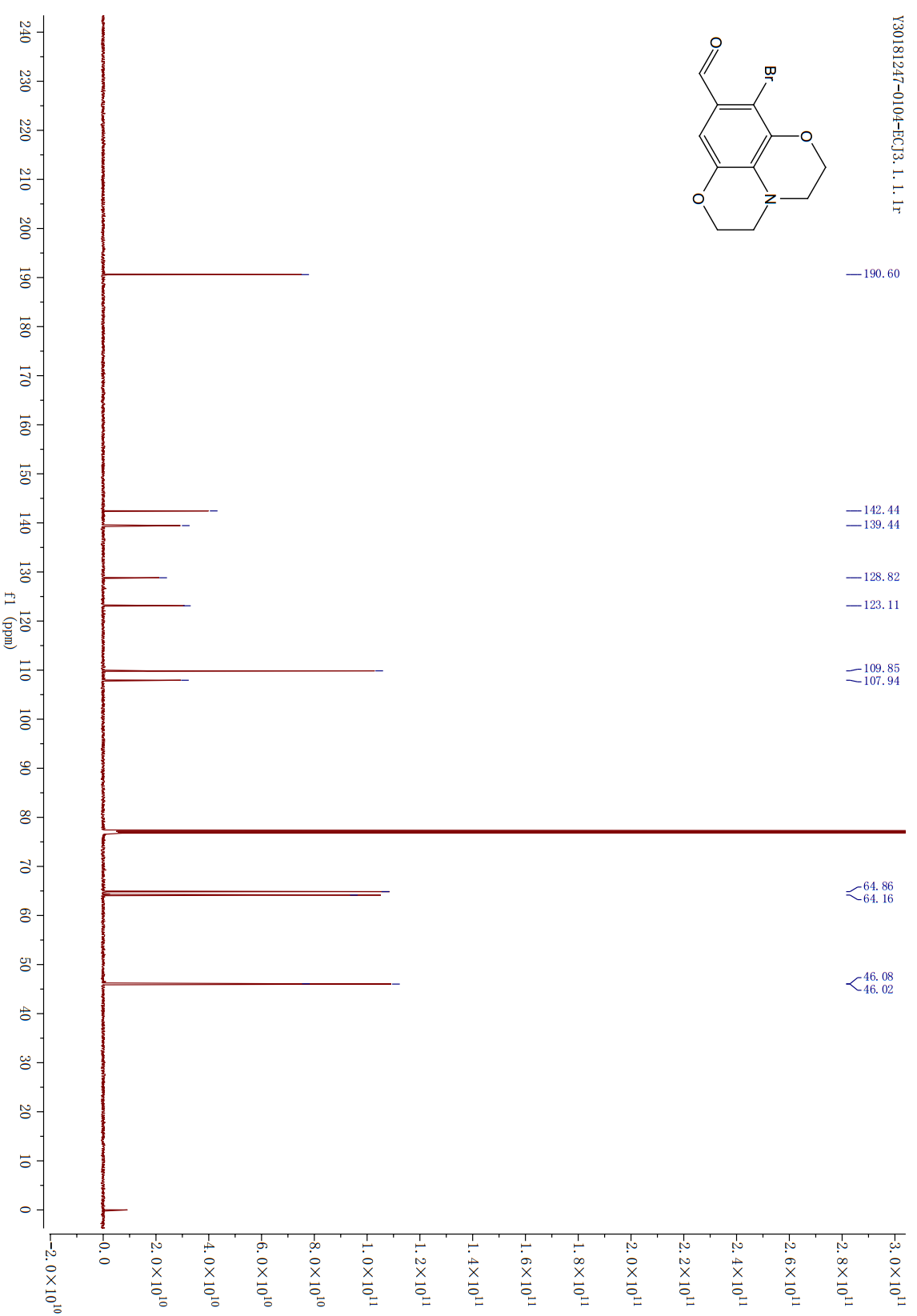


**Figure S15.** The ^13^C-NMR of compound **7** in CDCl_3_


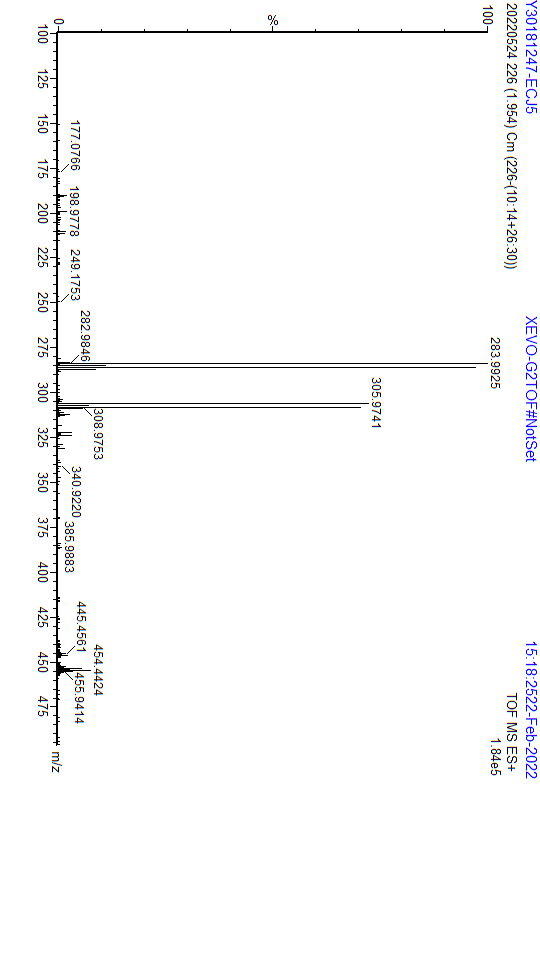


**Figure S16.** The HR-MS of compound **7**


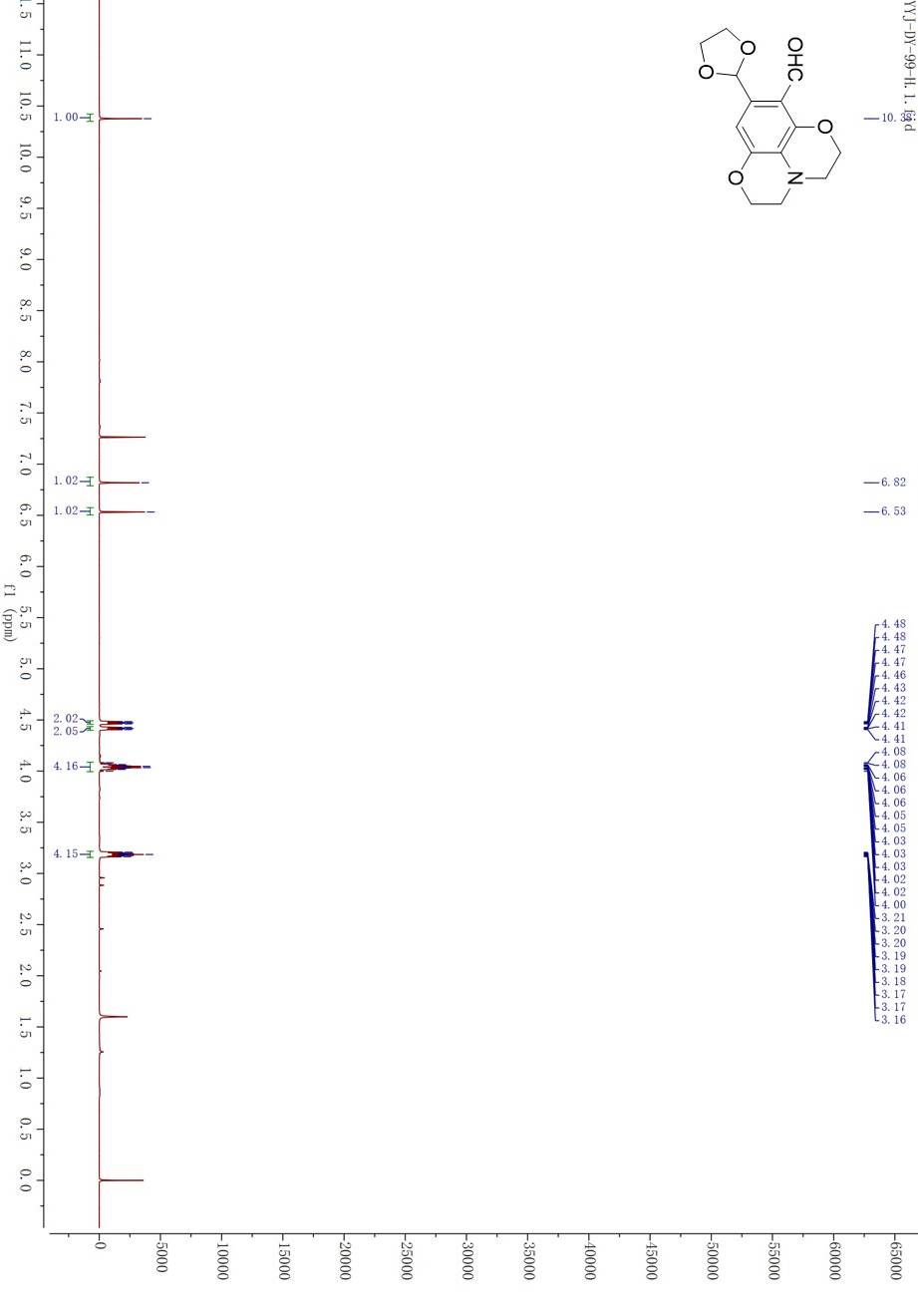


**Figure S17.** The ^1^H-NMR of compound **9** in CDCl_3_.


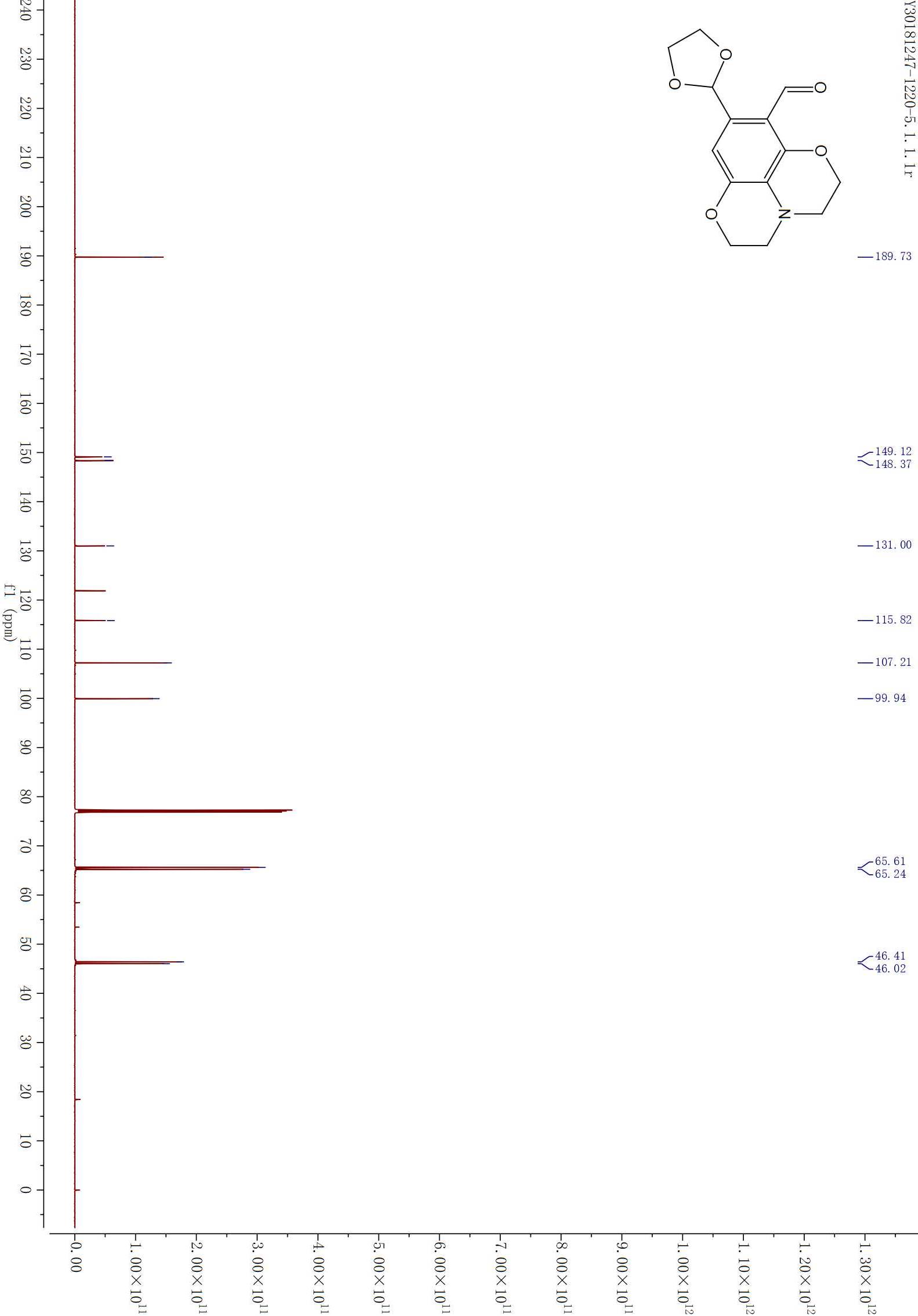


**Figure S18.** The ^13^C-NMR of compound **9** in CDCl_3_


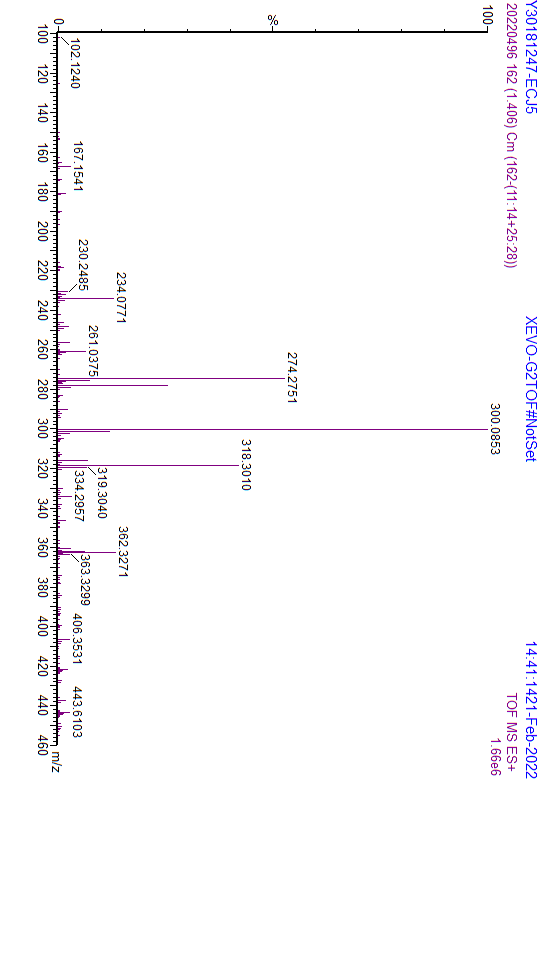


**Figure S19.** The HR-MS of compound **9**.


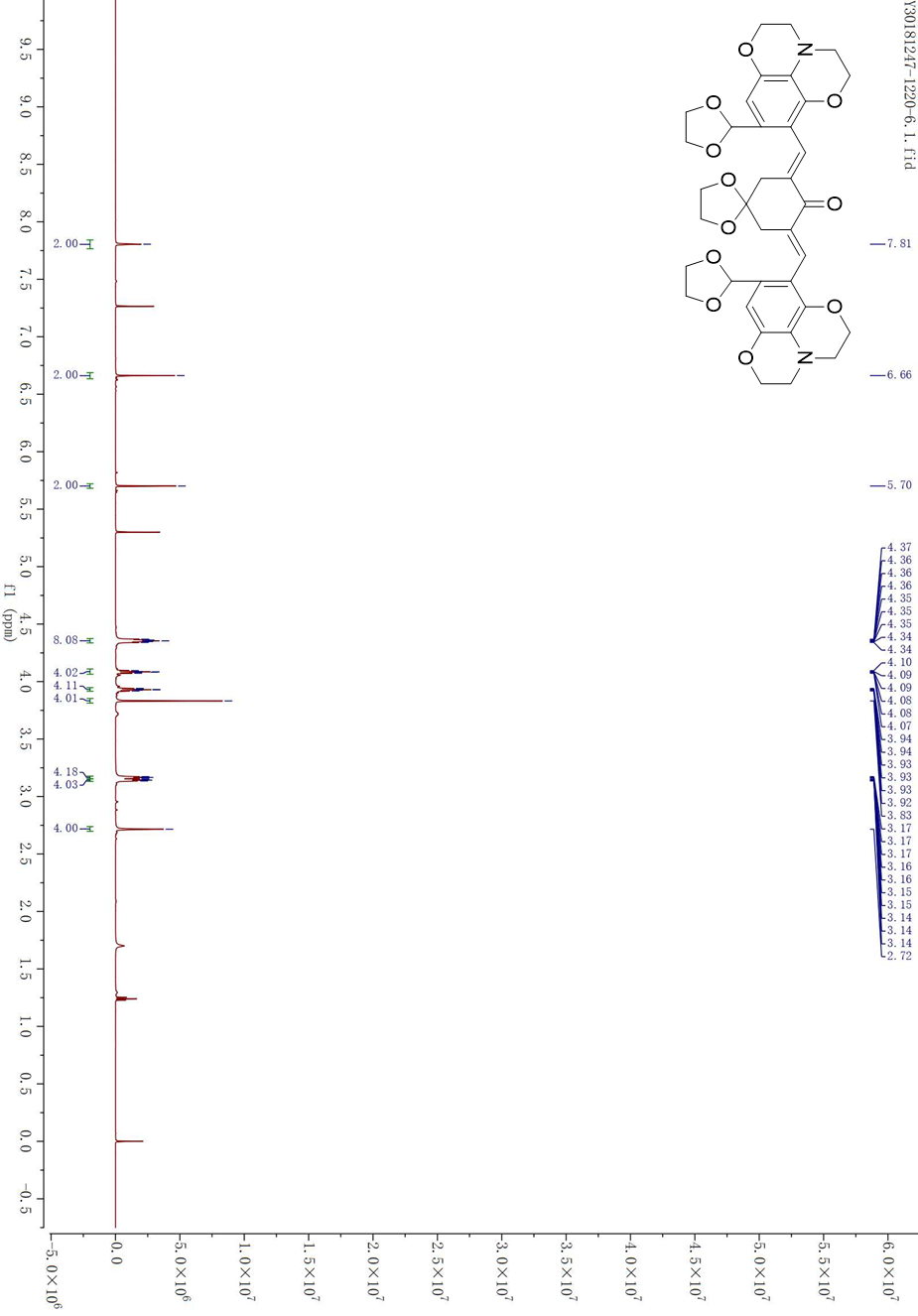


**Figure S20.** The ^1^H-NMR of compound **10** in CDCl_3_


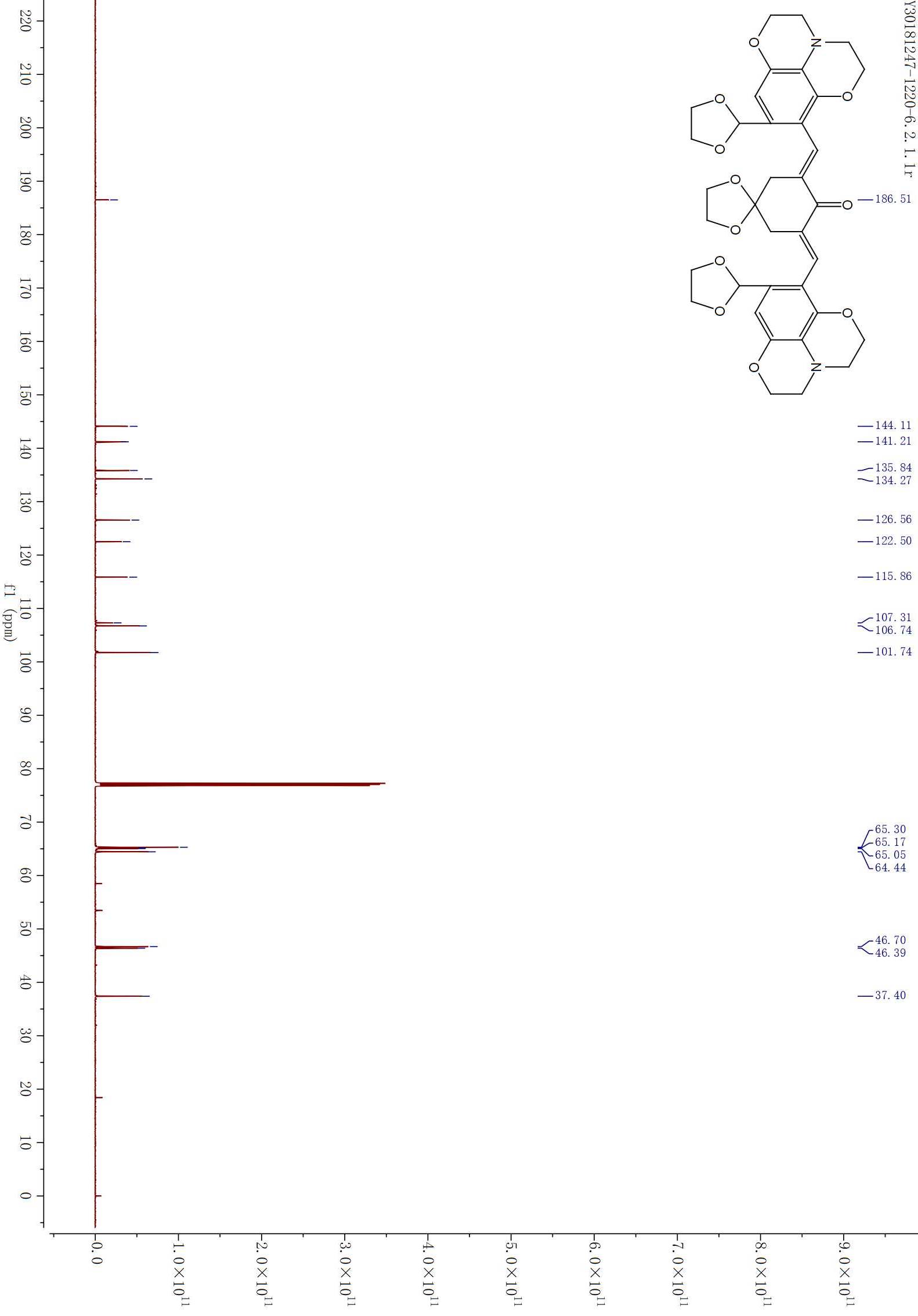


**Figure S21.** The ^13^C-NMR of compound **10** in CDCl_3_


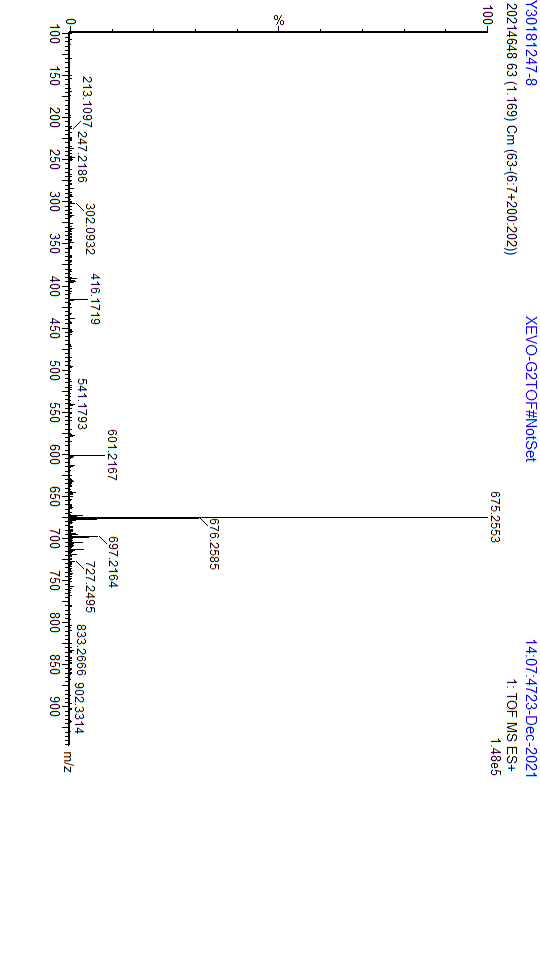


**Figure S22.** The HR-MS of compound **10**


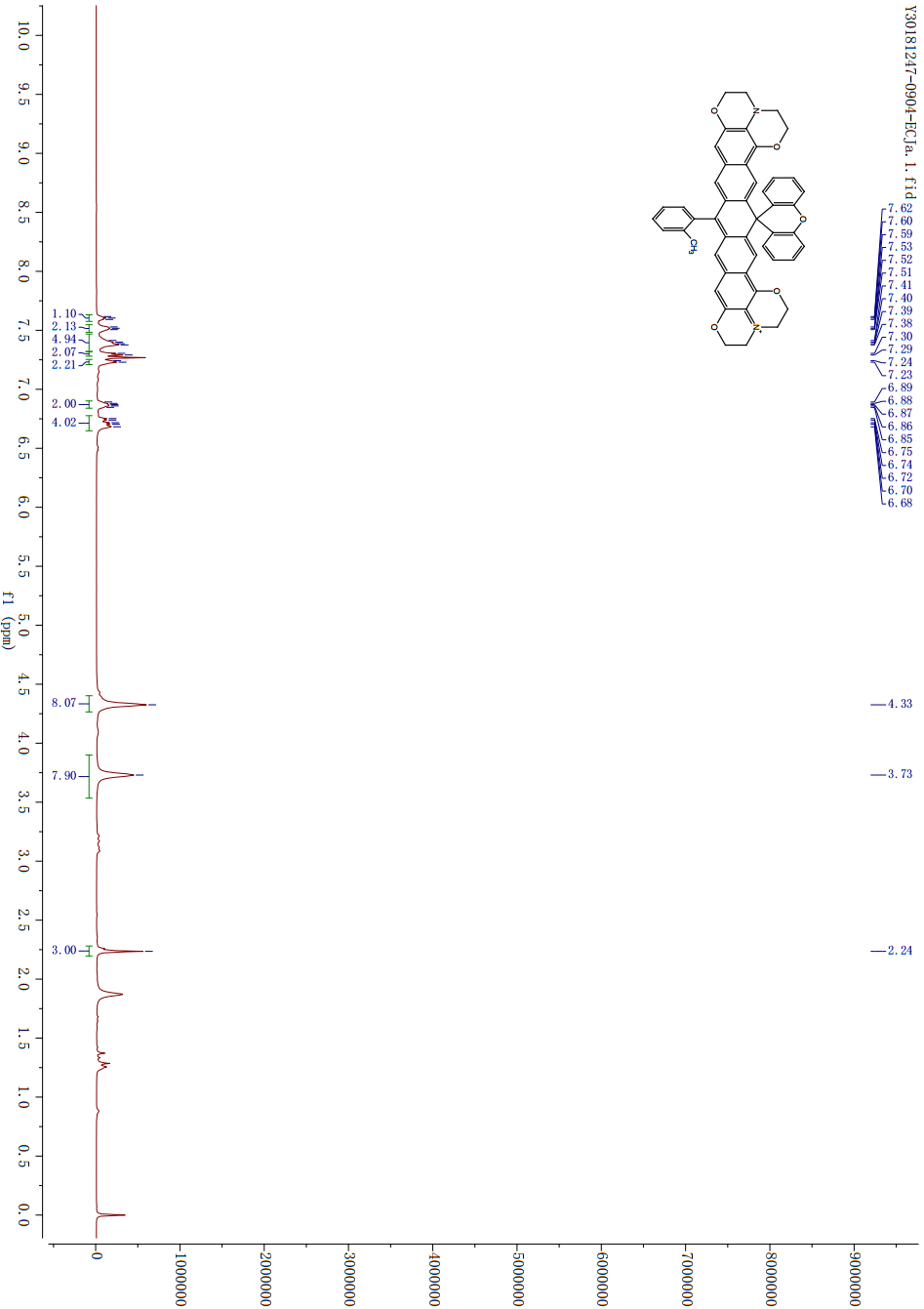


**Figure S23.** The ^1^H-NMR of compound **ECJ** in CDCl_3_

**Figure S24.** The ^13^C-NMR of compound **ECJ** in CDCl_3_


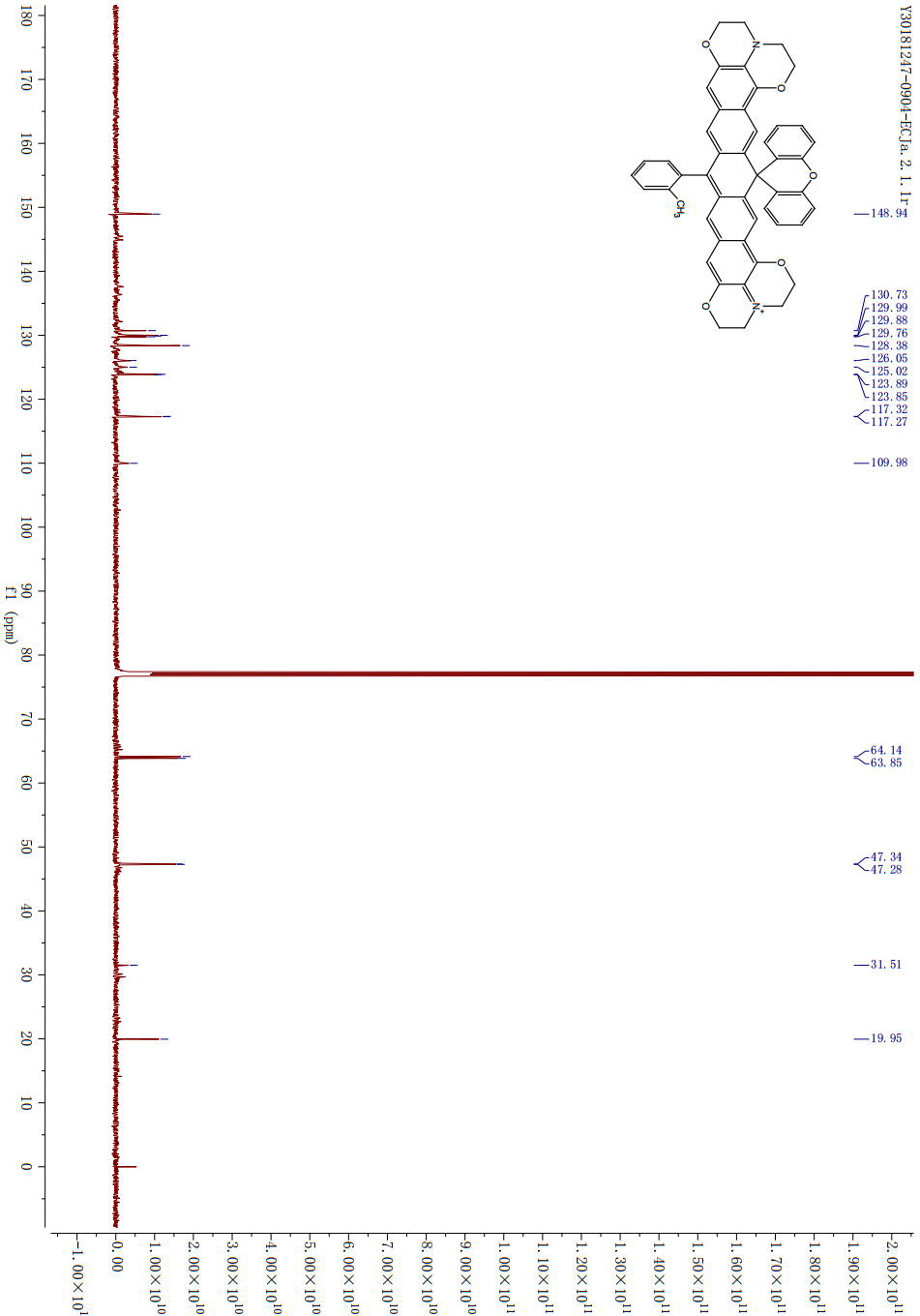


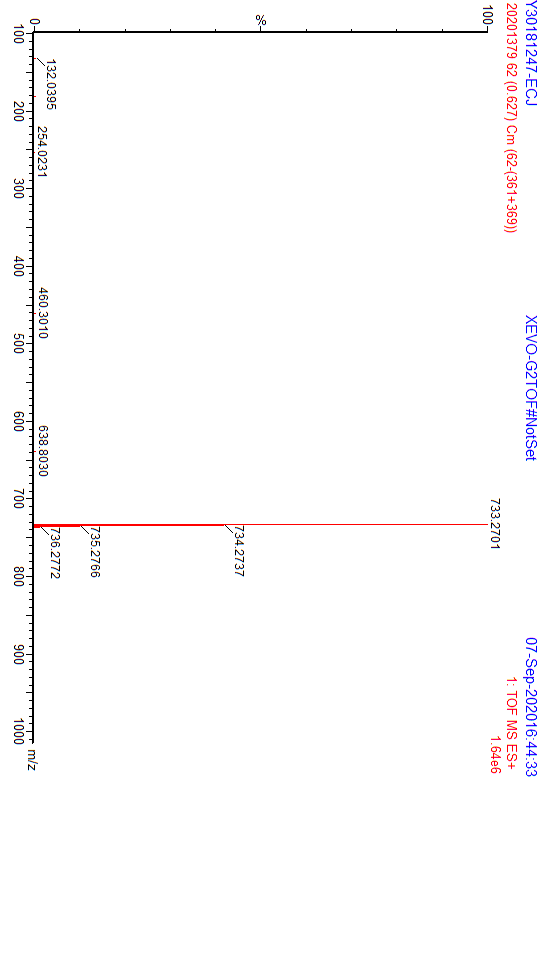
 **Figure S25.** The HR-MS of compound **ECJ**


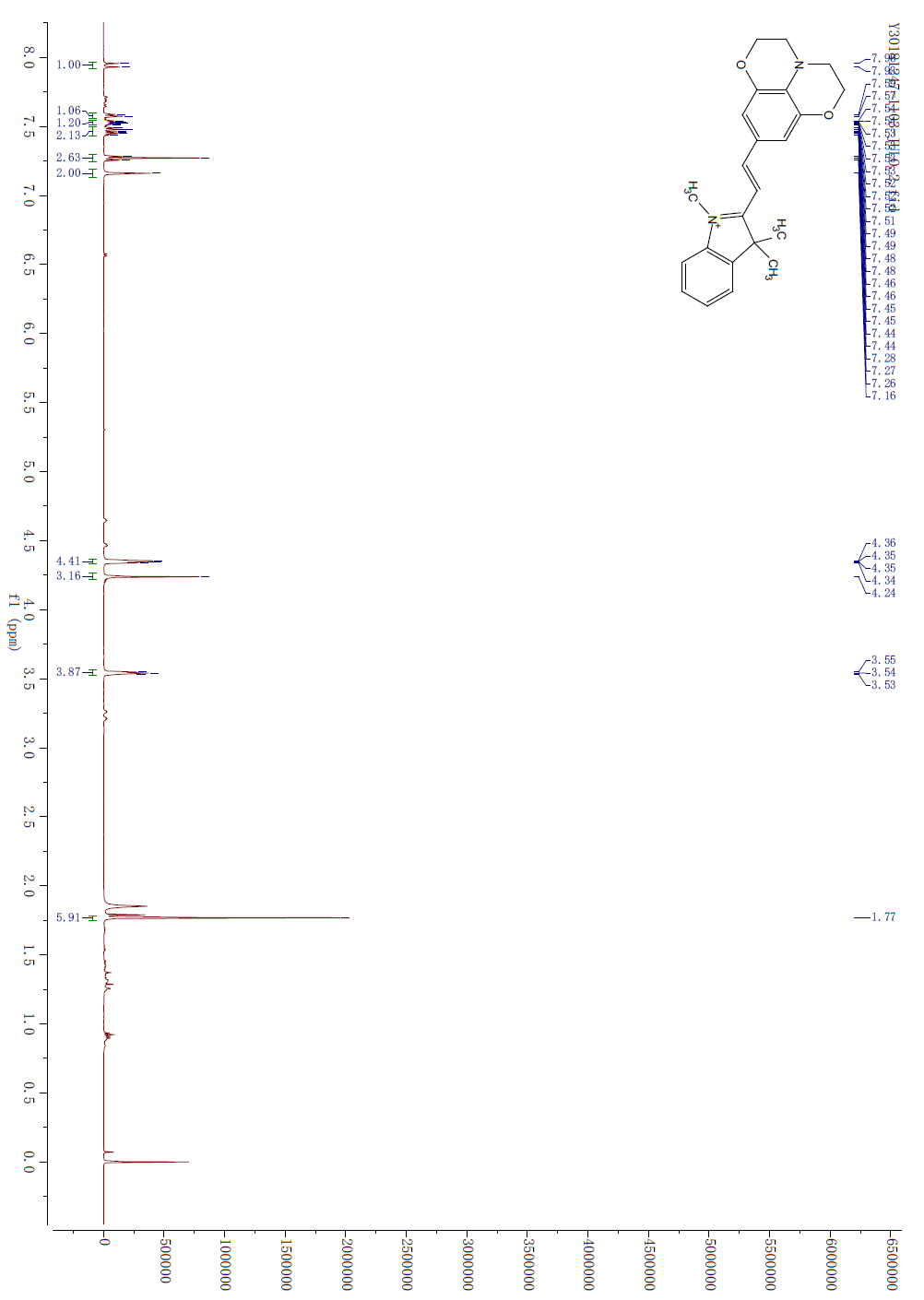


**Figure S26.** The ^1^H-NMR of compound **St6** in CDCl_3_


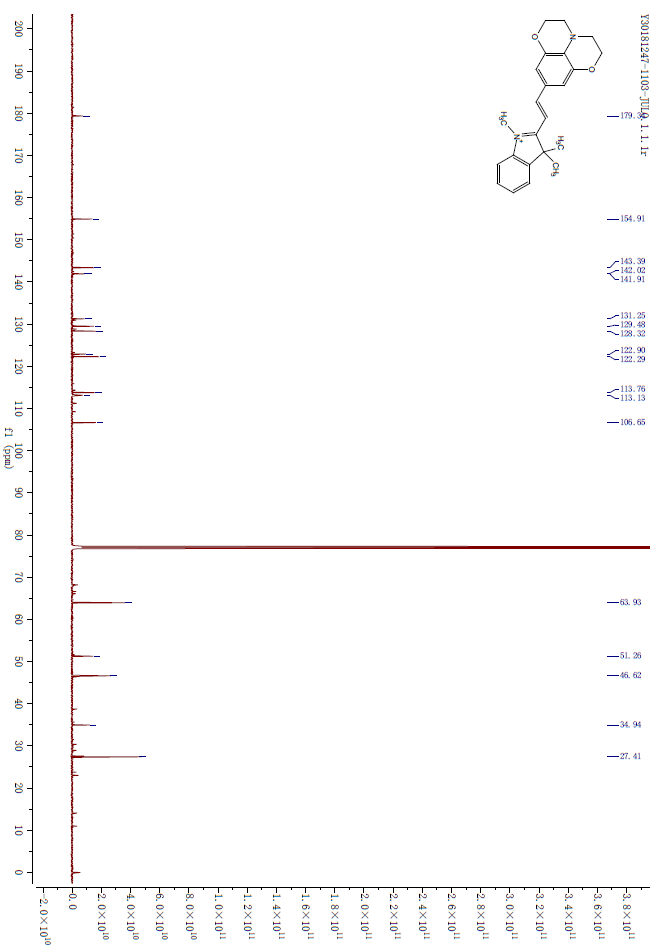


**Figure S27.** The ^13^C-NMR of compound **St6** in CDCl_3_


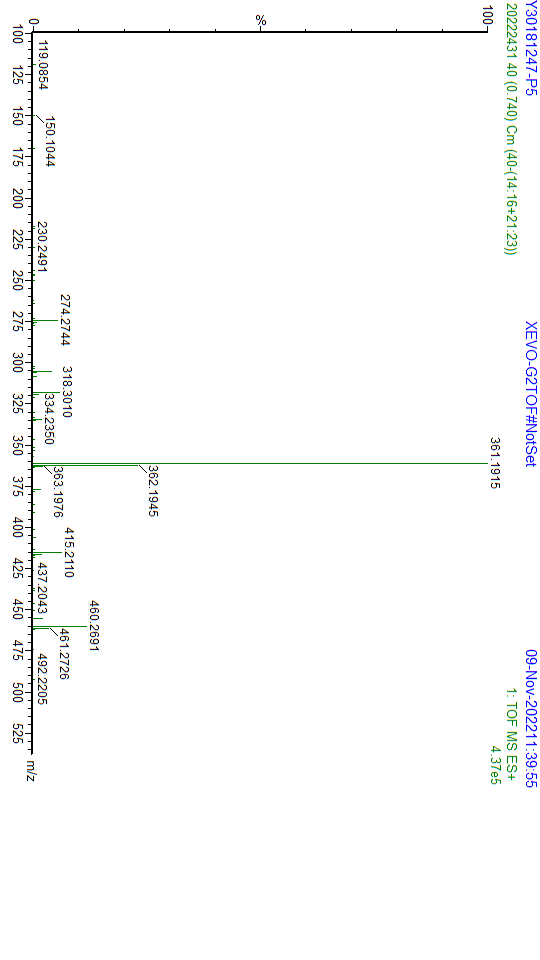


**Figure S28.** The HR-MS of compound **St6**


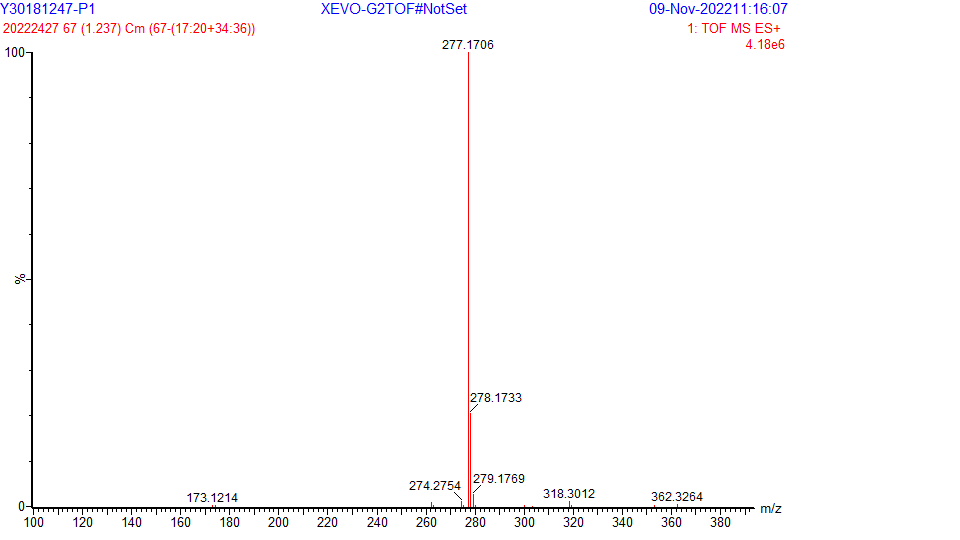


**Figure S29.** The HR-MS of compound **St1**


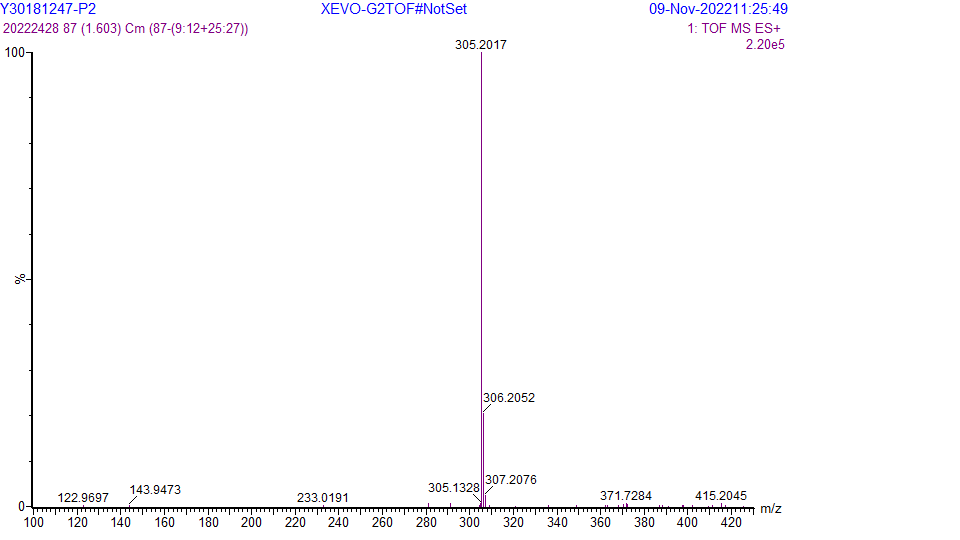


**Figure S30.** The HR-MS of compound **St2**


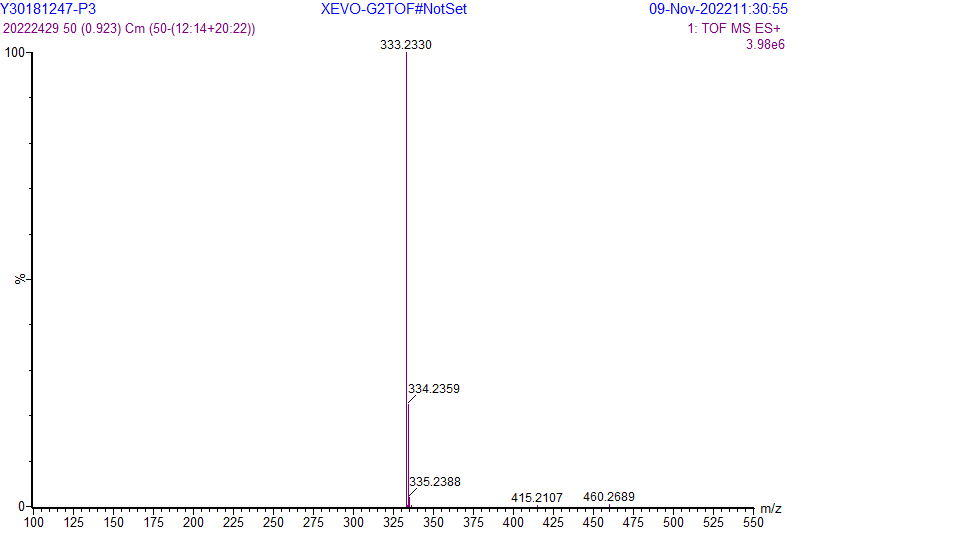


**Figure S31.** The HR-MS of compound **St3**


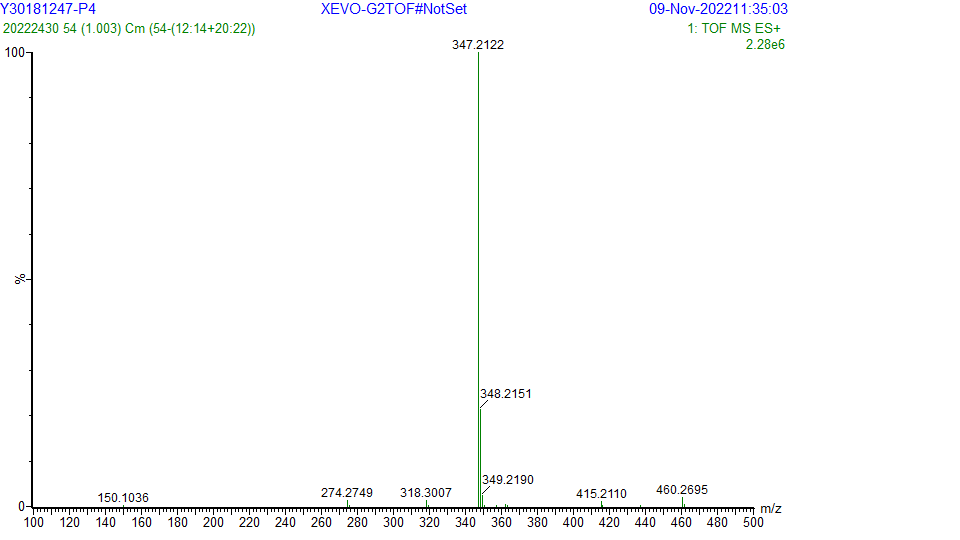


**Figure S32.** The HR-MS of compound **St4**


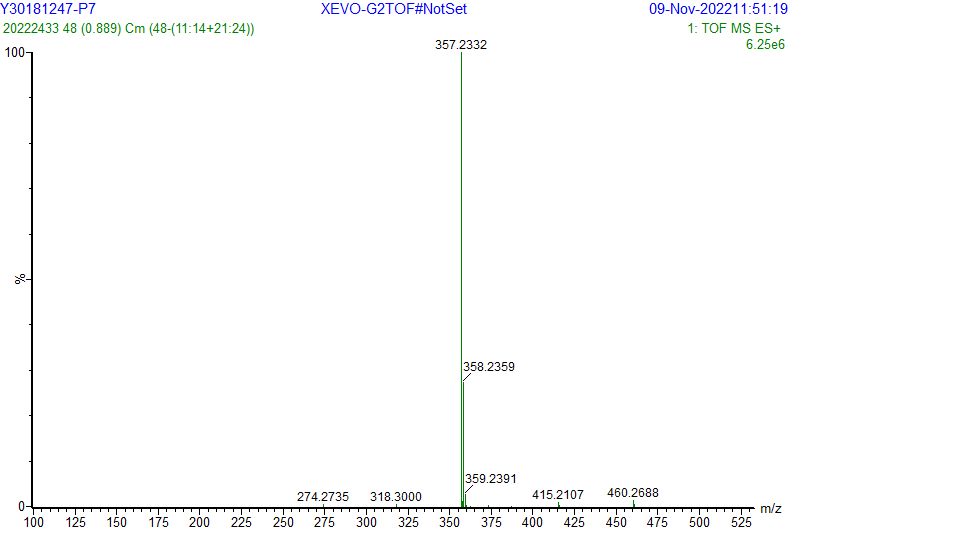


**Figure S33.** The HR-MS of compound **St5**

1. ^*^ Corresponding authors.

   *E-mail addresses*: [youjunyang@ecust.edu.cn](mailto:youjunyang@ecust.edu.cn) (Y. Yang). [↑](#footnote-ref-1)
